# Mapping current research on biomarkers associated with the diagnosis of pedophilia: a scoping review

**DOI:** 10.3389/fpsyt.2025.1627198

**Published:** 2025-10-16

**Authors:** Maria Karanikola, Anna T. El Riz, Andreas Chatzittofis

**Affiliations:** ^1^ Nursing Department, School of Health Sciences, Cyprus University of Technology, Limassol, Cyprus; ^2^ Medical School, University of Cyprus, Nicosia, Cyprus; ^3^ Department of Clinical Sciences and Psychiatry, Umeå University, Umeå, Sweden

**Keywords:** biomarkers, pedophilia, genetics, neuroimaging, hormones, physiological biomarkers, behavioral biomarkers

## Abstract

**Background:**

Pedophilia remains a challenging area of study due to its sensitive nature and the ethical considerations surrounding research involving individuals with deviant sexual interests.

**Objective:**

The aim of this review was to systematically explore and present the current research status on biomarkers in pedophilia. The focus was on biomarkers that may support the diagnostic process, treatment evaluation and assessment of risk and recidivism of pedophilia.

**Methods:**

Based on literature searches [MEDLINE, EMBASE, Scopus, APA PsycNet, Google Scholar], a scoping review was applied between January and March 2024, including studies in adults diagnosed with pedophilia, published within the last decade.

**Results:**

A total of 39 studies were included in the study sample. These encompassed only male participants. Biomarkers associated with pedophilia were identified and categorized as following: genetic/epigenetic and neuroendocrinal, physiological, cognitive/behavioral, and neuroimaging/neurofunctional. Results indicated the presence of cognitive deficits or impairments, especially in memory and executive functions, significant structural and functional brain differences in neuroimaging, with evidence of altered connectivity, volume reductions, and abnormal brain activation patterns. Physiological biomarkers revealed altered physical traits, attentional control, and sexual arousal patterns in pedophilia, with neural responses suggesting dysfunction in prefrontal cortex and error processing areas. Lastly, genetic and neuroendocrine studies suggested a potential link between epigenetic alterations in the serotonergic and testosterone systems, with lower testosterone levels and signs of prenatal androgen exposure observed in pedophilic individuals.

**Conclusions:**

This review mapped the existing state of the art data in biomarkers in pedophilia, also supporting the existence of promising biological systems implicated in the pathophysiology of pedophilia, thus emphasized the need for further research in the field.

**Systematic review registration:**

https://osf.io/8v9wn, identifier https://doi.org/10.17605/OSF.IO/8V9WN.

## Introduction

Pedophilia is a psychiatric disorder characterized by a primary or exclusive sexual attraction to prepubescent children (1). It is important to distinguish that not all individuals who commit child sexual offenses have pedophilia, and conversely, not all individuals with pedophilia engage in sexual offenses. Pedophilia refers specifically to a sexual preference, whereas child sexual offending involves actual criminal acts against children, irrespective of the perpetrator’s sexual orientation; in contrast, sexual arousal toward children is a prerequisite for the diagnosis of pedophilia (1).

According to the International Classification of Diseases, 11^th^ Revision (ICD-11) (1), pedophilic disorder is “characterized by a sustained, focused, and intense pattern of sexual arousal—as manifested by persistent sexual thoughts, fantasies, urges, or behaviors—involving pre-pubertal children”. Additionally, the person must either act on their sexual preference or suffer distress due to their deviant sexual preference (1). Similarly, the Diagnostic and Statistical Manual of Mental Disorders, Fifth Edition (DSM-5) defines pedophilic disorder as “recurrent, intense sexually arousing fantasies, sexual urges, or behaviors involving sexual activity with prepubescent children” persisting for at least 6 months, also causing marked distress or interpersonal difficulties (2).

In psychiatry, where diagnoses are based primarily on behavioral descriptions (e.g., those outlined in the DSM or ICD), developing objective biomarkers presents significant complexities (1–5). Nevertheless, pedophilia remains a challenging subject to study due to its ethical sensitivity and the difficulties surrounding research in individuals with deviant sexual interests. Factors such as stigma, legal risks, and institutional barriers often limit access to participants and constrain study designs, all resulting in samples that are predominantly drawn from forensic or incarcerated populations (3). This can introduce bias and limit the generalizability of findings across the broader spectrum of individuals with pedophilic interests (1–3).

Understanding the neurobiological underpinnings of pedophilia and identifying reliable biomarkers is essential for improving diagnostic accuracy, guiding treatment, and assessing risk and recidivism (3, 4).

Data from systematic reviews and meta-analyses examining the psychological, emotional, and social impact of pedophilic assault on survivors and their families help contextualize the broader consequences of pedophilic behavior (6, 7). These findings not only underscore the public health relevance of the issue but also highlight the critical need for accurate diagnosis and early identification, areas where biomarker research may offer valuable tools. To further illustrate the scope of this impact, child sexual abuse, often perpetrated by individuals with pedophilic disorder, has profound and long-lasting effects on survivors and their family systems. An umbrella review by Hailes et al. (2019), found significant associations between childhood sexual abuse and a wide range of psychiatric, psychosocial, and physical health outcomes (6). The strongest associations were identified for conversion disorder, borderline personality disorder, anxiety, and depression. Beyond the individual survivor, empirical evidence also demonstrates the substantial emotional toll experienced by non-offending family members. The study by Fong et al. (2020) revealed that caregivers experienced significant emotional and psychological distress, including anger, depressive symptoms, and guilt (7). Sources of distress included concerns about their child’s well-being, negative self-perceptions regarding their parenting, family members’ reactions, and even resurfacing memories of their own past maltreatment. These findings emphasize the enduring impact of such abuse and reinforce the importance of early detection strategies, including biomarker research, to prevent or mitigate long term harm.

Biomarkers are defined as “a characteristic that is objectively measured and evaluated as an indicator of normal biological processes, pathogenic processes or pharmacological responses to a therapeutic intervention” (5, 8). Research has proposed a range of potential biomarkers for pedophilia, including neuroimaging, neurochemical indicators and physiological assessment, including penile plethysmography and eye tracking (3, 4, 9–11). Some studies have even explored genetic markers (4). However, many of these findings stem from small, cross-sectional studies, and replication remains limited (3, 4, 9–11). Furthermore, several proposed biomarkers lack specificity and may overlap with other psychiatric or developmental conditions, which complicates their interpretation in clinical contexts (1, 2).

It is also important to recognize that biomarkers are not intended to replace clinical judgment or comprehensive assessments. Rather, they should be viewed as potential supplements to existing diagnostic and risk-evaluation tools, contributing to a multi-modal understanding of the condition. Lastly, cultural context, societal stigma, and the highly sensitive nature of pedophilia continue to shape both the availability of research data and the ethical boundaries within which such studies must operate. Yet, conducting systematic reviews that synthesize existing evidence is crucial in identifying gaps, refining methodologies, and establishing a more comprehensive understanding of pedophilia biomarkers.

Thus, the aim of this scoping review was to explore and map the current research state of research on biomarkers for pedophilia. The focus was on biomarkers that may support diagnostic processes, treatment monitoring and assessment of risk and recidivism in individuals diagnosed with pedophilia.

## Methods

### Research questions

Based on the aim of the present scoping review, the following research questions framed the inclusion criteria, search strategy, and synthesis:

1. What types of biological markers have been investigated in relation to the diagnosis or understanding of pedophilia?
2. What methodological approaches have been used to study biomarkers in individuals with pedophilic disorders? What are the main limitations, challenges, and ethical considerations reported in the literature regarding biomarker research in pedophilia?
3. What are the reported structural, functional, genetic, cognitive, and physiological differences associated with pedophilia in the existing literature?
4. How do biomarkers differ between pedophilic individuals who have offended and those who have not?

### Protocol and registration

This scoping review followed the methodological framework proposed by current guidelines for conducting scoping reviews (12). Reporting adhered to the Preferred Reporting Items for Systematic reviews and Meta-Analyses extension for Scoping Reviews (PRISMA-ScR)^2^ checklist (please see **Table S1** in **Supplementary material**. The protocol was registered on Open Science Framework-OSF (https://osf.io/8v9wn).

### Eligibility criteria

Eligible studies were empirical, peer-reviewed publications, focused on adults diagnosed with pedophilia, including both offenders and non-offenders, irrespective of: (a) the setting or context in which diagnosis was established, (b) patient background characteristics (e.g., age, treatment status, comorbidities), and (c) diagnostic and reporting method used (e.g., clinical interviews, metric tools: questionnaires/scales, physical examination, or self-report).

Comparative studies evaluating interventions or diagnostic tools/procedures in the target population were also included.

Systematic reviews, meta-analyses, narrative reviews, case reports and case series, empirical studies with fewer than 10 participants, and conference abstracts, were excluded. Empirical studies exclusively including non-pedophilic sexual offenders and records in non-English were both excluded. Restrictions were applied regarding the year of publication to exclusively include state-of-the art research on biomarkers, e.g., published work in the last decade.

### Information sources

A comprehensive literature search was conducted from January to March 2024 by two experienced health researchers. The search strategy was developed in consultation with a medical librarian to optimize the rigor of the process. The databases searched included: MEDLINE via OVID, EMBASE via OVID; PsycINFO; Scopus via Elsevier.

### Search strategy

A combination of controlled vocabulary (MeSH terms) and free-text key-words vocabulary was employed. The MEDLINE strategy included terms as follows: ((‘child sexual abuse’[MeSH Terms] OR pedophilia[MeSH Terms] OR paedophilia[MeSH Terms]) AND (biology[Title/Abstract] OR biological[Title/Abstract] OR genetic[Title/Abstract] OR epigenetic[Title/Abstract] OR imaging[Title/Abstract] OR biomarkers[Title/Abstract] OR hormones[Title/Abstract] OR EEG[Title/Abstract] OR neuropsychological[Title/Abstract] OR neurotransmitters[Title/Abstract] OR ‘penile plethysmography’[Title/Abstract] OR ‘eye tracking’[Title/Abstract] OR etiology[Title/Abstract]).

Additional search methods included backward citations tracking and forward citation searching of relevant reviews and included articles.

### Selection of sources of evidence

Covidence tool (13) for data management, automatic duplicate removal and independent screening of relevant studies was used to export search outcomes. Two members of the research team (AC, AE) independently screened abstracts and titles of search outcomes for relevance. In cases that the relevance of a study was unclear according to the title or the abstract, the full text was reviewed. Any discrepancies were resolved through discussion or by consultation with a third researcher (MK).

### Data charting process

All search outcomes deemed as relevant were independently reviewed by the three researchers (MK, AE, AC) to confirm eligibility. This was achieved by fully assessing the scope of data responding to the objectives of the present review. Inter-rater reliability was monitored and documented at this point, by the degree of agreement between the three researchers, regarding the inclusion or not of all search outcomes deemed as relevant. A custom data assessment tool (“include”/”exclude with documentation comments”) was developed and pilot tested on six initial studies.

Data extraction was guided by a standardized form created by AC and tested by AE. Data elements and extraction methods were mutually agreed upon by all members of the research team. Extracted data included: first author, country of study, year of publication, study design and setting, sample characteristics (sampling method, sex, size, age), diagnostic methods and inclusion criteria, primary result on biomarkers, biomarker type/category, and testing paradigm/methods.

### Critical appraisal of individual sources of evidence

The search outcomes which were confirmed as relevant underwent methodological quality assessment using the Joanna Briggs Institute critical appraisal tools (14). Three researchers (MK, AE, AC) independently conducted the appraisal, and discrepancies were resolved according to the degree to which the researchers identified supporting data in the manuscript for those items identified as controversial. An assessment form was created to document assessment judgments. These data are presented in **Table S2**, **S3** in **Supplementary material**. Although methodological quality was appraised, no studies were excluded based on quality to preserve the breadth of data, consistent with scoping review methodology. Specifically, the aim of a scoping review is to present the spectrum of existing data on a topic, along with the quality of this data instead of focusing on the relationship among variables and relevant effect size (15).

### Synthesis of results

The synthesis of data involved narrative aggregation and mapping of identified biomarkers across studies. Key patterns, trends, and methodological limitations were summarized to capture the current state of evidence and to highlight gaps. This synthesis provided insight into the diversity and limitations of the evidence base and suggested directions for further biomarker research in pedophilia.

## Results

### Selection of sources of evidence

**Figure 1:**
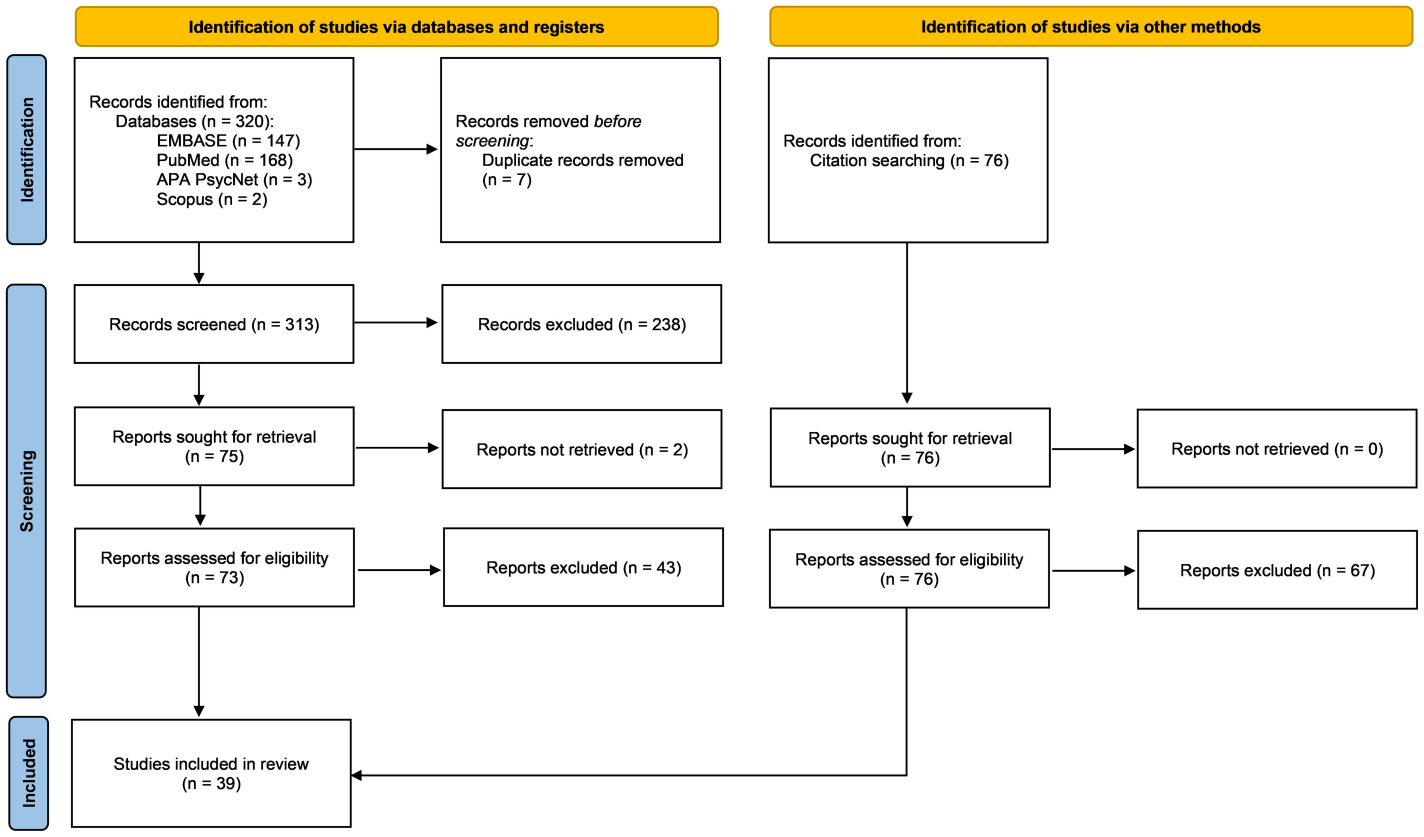
Prisma flow chart of the identification and inclusion/exclusion process.

A total of 320 studies were identified through four databases: EMBASE (n=147), Medline (n=168), PsycINFO (n=3), and Scopus (n= 2). After the removal of duplicates, 313 titles and abstracts were screened. Of these, 238 were excluded, 75 were sought for retrieval and 73 were reviewed in full for eligibility (two studies could not be accessed in full text). An additional 43 studies were excluded based on eligibility criteria. Nine additional studies were identified through hand-searching, resulting in 39 studies included in the review (please see **Figure 1**).

### Characteristics of sources of evidence

Details of the 39 included studies, such as author, year of publication, country of origin, research aim/objectives, sample characteristics, methodology, are presented in **Tables 1**–**4**. All studies were published between 2013 and 2023 in English. Most were conducted in Germany (n=23), followed by Canada (n=5), and the U.S.A (n=3). Twelve studies recruited participants from the “Neural Mechanisms Underlying Pedophilia and Sexual Offending Against Children” (NeMUP) research project. The majority were case-control studies (n=37), with two cross-sectional studies (n=2). Samples typically required participants to be aged 18 years or older, and diagnosed with pedophilia, including both offending and non-offending exclusively male individuals.

**Table 1:** Study characteristics and main findings on neuroimaging/neurofunctional biomarkers.

Neuroimaging/Neurofunctional biomarkers						
No	Authors, year	Origin of study	Aim/Outcome	Sample type, size and mean age (SD)	Testing methods	Findings
1	Abé et al. (2021) (16)	Sweden	To identify clinically useful biomarkers and risk factors by investigating PD-related neurobiology using magnetic resonance imaging (MRI), psychiatric assessment, and cognitive testing.	55 self-referred, help-seeking, non-forensic male patients with DSM-5 PD were recruited through PrevenTell, a Swedish national helpline, 57 age-matched, healthy male controls (HC) screen-negative for PD, were recruited in the Stockholm catchment area through Karolinska Trial Alliance and adverts on Karolinska Institute's homepage. PD: M=36 (12), HC: M=36 (12)	Psychiatric Comorbidity & Clinical Symptoms: M.I.N.I. 6.0.0 for coexisting psychiatric disorders, RAADS-14 for autism spectrum disorder, ASRS-v1.1 for ADHD symptoms. General cognitive ability: WAIS-IV. Anthropometric measurements: participants’ height, weight, lengths of index finger (2nd digit) and ring finger (4th digit) of the right hand for assessment of the 2D:4D digit ratio. Structural MRI image acquisition and processing	• PD patients had lower total IQ than HC. • PD patients had moderately and significantly smaller intracranial volume. • PD showed smaller cortical surface area in clusters comprising (i) bilateral ventromedial prefrontal cortex, (ii) caudal and posterior cingulate cortex, precuneus and paracentral cortex, supramarginal and superior/inferior parietal cortex, (iii) left fusiform/inferior temporal and lingual cortex, (iv) middle and superior temporal cortex, (v) insula, lateral prefrontal cortex (pars opercularis/triangularis, caudal/rostral middle frontal), extending into pre- and postcentral cortex. • No group differences in cortical thickness. • PD individuals exhibited cortical surface area abnormalities in regions belonging to the brain's default mode network and showed abnormal volume of white matter in regions where surface abnormalities were found. • PD subjects had smaller hippocampi and nuclei accumbens than HC.
2	Cantor et al. (2015) (17)	Canada	To provide more precise characterization of cerebral white matter among pedophiles.	Pedophilic sex offenders (n=24) were compared with healthy, age-matched controls with no criminal record and no indication of pedophilia (n=32). Sexual offender participants were recruited from the Kurt Freund Laboratory of CAMH. Controls were recruited from an online bulletin board. Pedophiles: M=35.63 (9.52), Controls: M=37.00 (10.72)	Tract-Based Spatial Statistics (TBSS), probabilistic tractography, diffusion tensor imaging (DTI), the Shipley Institute of Living Scales, Edinburgh Handedness Inventory, modified version of the Conflict Tactics Scale, the Self-Reported Childhood Abuse—Physical scale, the Childhood Neglect Index, the Widom Child Sexual Abuse Interview, the Levenson Psychopathy Scale, the CAGE alcohol use screening instrument, and the Structured Clinical Interview for DSM-IV, Phallometric Pedophilia Index (PPI), Voxel-Based Morphometry (VBM)	• Groups showed significant, highly focused differences in DTI parameters which related to participants’ genital responses to sexual depictions of children, but not to measures of psychopathy or to childhood histories of physical abuse, sexual abuse, or neglect. • Group differences in several gray matter structures were suggested under highly liberal statistical conditions (p_uncorrected_<.005), but did not survive ordinary statistical correction. • Fractional anisotropy (FA) across the cluster was significantly elevated in the pedophilic group. These parameters reflected significantly greater axial diffusivity and significantly lower radial diffusivity. • FA significantly correlated with penile responsivity to erotic depictions of children. • Probabilistic tractography identified the endpoints/sources of the group differences in connectivity to be insula/operculum, superior temporal gyrus, temporal pole, occipital cortex, dorsolateral prefrontal cortex, temporal–occipital junction, superior parietal lobule, frontal pole, and thalamus (all unilateral left).
3	Cantor et al. (2016) (18)	Canada	To identify analogous differences in functional connectivity.	A group of 37 pedophilic men and two types of control group: 28 non-pedophilic men who committed at least one non-sexual offense and 39 non-pedophilic non-offenders. Pedophilic men were recruited from the Kurt Freund Laboratory of the Centre for Addiction and Mental Health. The two control groups were recruited from an online bulletin board. P+CSO: M=35.70 (1.70), Non-Pedophilic+Non-Sexual Offense: M=40.50 (1.69), HC: M=35.95 (1.73)	Phallometric Pedophilia Index, the Structured Clinical Interview for DSM-IV, the Shipley Institute of Living Scales, the Edinburgh Handedness Inventory, the CAGE alcohol use screening instrument, the Levenson Psychopathy Scale, the Conflict Tactics Scale, the Self-Reported Childhood Abuse—Physical scale, the Childhood Neglect Index, the Widom Child Sexual Abuse Interview, a current and lifetime drug use questionnaire, and a questionnaire of history of head trauma or neurologic disease, MR Image Acquisition	• The pedophilic group demonstrated wide-ranging increases in functional connectivity with the default mode network compared with controls and regional differences (increases and decreases) with the frontoparietal network. • Of these brain regions (total =23), 20 have been identified by meta-analytic studies to respond to sexually relevant stimuli. • Conversely, of the brain areas known to be those that respond to sexual stimuli, nearly all emerged in the present data as significantly different in pedophiles.
4	Cazala et al. (2019) (19)	France	To identify through functional magnetic resonance imaging the brain responses of patients with pedophilic disorder to visual stimuli depicting children (VSc) and to compare them with matched healthy controls.	25 male outpatients with pedophilic disorder, 24 male healthy controls matched on sexual orientation (to female or male adults), age, and handedness were recruited through a web platform dedicated to informing potential participants about current investigations in psychology and neuroscience in the Parisian region. Inclusion criteria common to the two groups were male gender and age between 18 and 65. Patients: M=42.2 (12.0), Controls: M=37.8 (12.8)	Handedness using Edinburgh Handedness Inventory, IQ using Wechsler Adult Intelligence Scale-III, Sexual function using Sex History Questionnaire Revised (SHQ-R), the Sexual Fantasies, Desires and Activity Interview Schedule (SFDAIS), the Sexual Interest Score (SIS), the Brief Sexual Function Questionnaire (BSFQ), the Sexual Arousal Inventory (SAI), hormonal investigation using a blood sample (testosterone, sex-hormone binding globulin SHBG, LH, FSH, prolactin, and cortisol), fMRI using different categories of pictures (stimuli), plethysmographic data using penile plethysmography, behavioral and endocrine data	• No region was differentially activated across the two groups in response to visual stimuli depicting children (VSc). In patients with pedophilia, the presentation of VSc induced a bilateral activation in the lateral occipital and temporal cortices, (right inferior temporal gyrus) • In patients the level of bilateral activation in the above-mentioned regions was positively correlated with ratings of perceived sexual arousal elicited by VSc. • In patients the blood-oxygen-level-dependent (BOLD) level in these areas was positively correlated with the ratings of Desire for Sexual Activity (DSA) and of Perceived Erection (PE) • Greater correlation in patients than in controls between the left posterior cingulate activation and the ratings of PE was related to the high correlation found only in patient
5	Fonteille et al. (2019) (20)	France	To identify through positron emission tomography the brain responses of males with pedophilic disorder to validated visual sexual stimuli depicting children (VSSc) and to compare them with male healthy controls matched for sexual orientation (to female or male adults), age, and handedness.	15 male outpatients with pedophilic disorder, 15 male healthy controls matched for sexual orientation, age, and handedness. Patients were recruited through centers specialized in the treatment of persons with sexual offense histories and of patients with paraphilias. Controls were recruited through a web platform dedicated to informing potential participants about current investigations in psychology and neuroscience. Patients: M=42.0 (12.6), Controls: M=41.2 (16.2)	DSM-IV-TR, ICD-10, the Edinburgh Handedness Inventory score, of the Wechsler Adult Intelligence Scale-III, the Sex History Questionnaire Revised SHQ-R, questionnaire on Sexual Fantasies, Desires and Activity (SFDAQ), the Brief Sexual Function Questionnaire (BSFQ), the Sexual Arousal Inventory (SAI), the Symptom Checklist-90-Revised (SCL-90R), the 21-item Beck Depression Inventory version, the Barratt Impulsiveness Scale (BIS-10), sex-hormone binding globulin (SHBG), bioavailable testosterone (not bound to SHBG), estradiol, LH, FSH, prolactin, cortisol, PET scan, volumetric penile plethysmograph	• Compared with controls, patients showed a higher difference between Desire for Sexual Arousal (DSA) ratings for undressed and for normally dressed children. The same Group by Condition interaction was found for erectile responses to VSSc. • In response to VSSc, the between-groups analysis showed that activation in the right inferior temporal cortex [Brodmann area (BA) 20] was lower in patients than in controls. • In patients but not in controls, the presentation of VSSc induced an activation in a more caudal region of the right inferior temporal gyrus (BA 37) and in the left middle occipital gyrus (BA 19). • In patients the level of activation in the caudal right inferior temporal gyrus was positively correlated with ratings of sexual arousal elicited by VSSc, whereas this correlation was negative in BA 20.
6	Gerwinn et al. (2015) (21)	Germany	To search for gray and white matter differences between pedophilic and teleiophilic subjects.	24 pedophilic and 32 teleiophilic men. Subjects were partially recruited from two outpatient facilities of a child sexual abuse prevention project “Dunkelfeld” for self-acknowledged pedophiles. The control group of teleiophilic men, recruited 18 gynephilic (HeTe) and 14 androphilic (HoTe) volunteers not significantly different from the pedophilic group in terms of sexual gender orientation, handedness, age and IQ. Mean age: NOT PROVIDED	Voxel-based morphometry (VBM) of the T1-weighted images and tract-based spatial statistics (TBSS), Diffusion tensor imaging	• No significant gray or white matter differences were found between the pedophilic and teleiophilic subjects, after adjustment for multiple comparisons and controlling for important confounding factors.
7	Gibbels et al. (2019) (22)	Germany	To carefully analyze clinical, neuropsychological and neurobiological features of convicted and non-convicted CSOs to unravel possible factors that might increase or decrease the probability of being convicted.	Overall, 31 non-convicted men CSOs and 48 men convicted CSOs. Participants were recruited as part of the multi-site research project NeMUP study. Some subjects were recruited in prisons or during fulfillment of a suspended sentence. The analysis of the Go/No-go paradigm combined with fMRI included 23 non-convicted CSOs and 38 CSOs (not all participants fulfilled the criteria to be examined with MRI). Non-Convicted CSOs (n = 31): M=40.4 (10.8), Convicted CSOs (n = 43): M=39.8 (9.0)	MRI, DSM structured clinical interview (SCID), intelligence using WAIS-IV, empathy using multifaceted empathy test (MET), impulsivity Barratt impulsiveness scale (BIS-11), Go/No-go task	• Successful response-inhibition activated clusters in the anterior cingulate cortex, supplemental motor cortex, insula and middle temporal gyrus in both groups with n differences between them.
8	Habermeyer et al. (2013) (23)	Switzerland	To address inhibition behaviorally and by means of functional imaging, aiming to assess how inhibition in pedophilia is related to a differential recruitment of frontal brain areas.	18 male right-handed subjects: 11 pedophilic subjects, 7 non-pedophilic controls. Pedophilic subjects (n = 11) were recruited from an outpatient cognitive behavioral group therapy at the Forensic Psychiatric Hospital, Basel, Switzerland. Seven control subjects were recruited using an advert on the University Hospital bulletin board. Pedophiles: M=49 (12.5), Controls: M=47 (8.6)	Go/NoGo Task, MRI scanner, Multiphasic Sex Inventory, revised German version of the Wechsler Adult Intelligence Scale	• Pedophilic subjects showed a slower reaction time and less accurate visual target discrimination. • fMRI voxel-level ANOVA revealed as a main effect of the go/no-go task an activation of prefrontal and parietal brain regions in the no-go condition, while the left anterior cingulate, precuneus and gyrus angularis became more activated in the go condition. • A group × task interaction was found in the left precuneus and gyrus angularis. This interaction was based on an attenuated deactivation of these brain regions in the pedophilic group during performance of the no-go condition. • The positive correlation between blood oxygen level-dependent imaging signal and reaction time in these brain areas indicates that attenuated deactivation is related to the behavioral findings.
9	Kärgel et al. (2015) (24)	Germany	To compare functional connectivity at rest (RSFC) between pedophiles who engaged or did not engage in CSA and healthy controls within two networks: (i) the default mode network and (ii) the limbic network	12 P+CSA,14 P–CSA and 14 HCs, all males. Pedophiles without a history of CSA were exclusively recruited from the darkfield, via short subject information texts in relevant Internet forums. Those with a history of CSA were recruited both via Internet forums and by correctional services in North Rhine-Westphalia, Germany. The HC group was recruited from the community throughout advertisements in public institutions. P+CSA: M=43.67 (7.08), P-CSA: M=28.07 (5.71), HC: M=32.86 (9.89)	DSM-IV-TR, Kinsey scale for developmental stages, German version of the Wechsler Adult Intelligence Scale, 4th edition (WAIS), MRI	• Pedophiles who engaged in CSA show diminished RSFC in both networks compared with HC and P–CSA. • They showed diminished RSFC between the left amygdala and orbitofrontal as well as anterior prefrontal regions.
10	Kargel et al. (2017) (25)	Germany	To analyze inhibitory control capacity and underlying inhibition- related neural activation pattern with respect to pedophilia and/or sexual offending against children, using event related fMRI in combination with a behavioral go/nogo paradigm.	117 men matched for age and IQ: 40 P+CSO, 37 P-CSO, 40 HC. Part of the (NeMUP) “Neural Mechanisms Underlying Pedophilia and Sexual Offending Against Children” project. P-CSO were recruited from the community (n=20) or the “Prevention Project Dunkelfeld” (PPD) (n=17). P+CSO were taken from the community (n=16), and the PPD (n=9), and from correctional institutions (n=15). The healthy control group recruited from the community throughout advertisements in public institutions included 40 men without a history of criminal behavior or current psychiatric disorders. P+CSO: M=38.25 (8.54), P-CSO: M=37.00 (8.84), HC: M=36.65 (10.13)	Go/No-Go paradigm, the Structured Clinical Interview for the DSM-IV-TR (SCID), modified version of the Kinsey scale for developmental stages, the German version of the Wechsler Adult Intelligence Scale, 4th Edition (WAIS-IV), MRI scanners.	• As compared to offending pedophiles, non-offending pedophiles exhibited superior inhibitory control as reflected by significantly lower rate of commission errors. • Pedophiles revealed decreased activation of the medial parietal cortex including the left caudal posterior cingulate cortex as well as the left superior frontal cortex as compared to non-offending pedophiles, but no activation difference in prefrontal areas, while no significant differences were found between pedophiles and healthy controls.
11	Lett et al. (2018) (26)	Germany	(1) To assess structural brain morphology using MRI-based analysis approaches such as whole brain analyses of cortical thickness (CT), surface area (SA), and white matter fractional anisotropy (FA), (2) to differentiate between P+CSO, pedophiles who have not committed CSO (P-CSO) and non-pedophilic controls (NPC) in a large neuroimaging sample, and (3) to examine a potential neurobiological mechanism in which intelligence may mediate the association between aberrant cortical morphology and CSO behavior.	73 P+CSO, 77 P-CSO, 133 non-pedophilic controls (NPC). Male participants were recruited within the NeMUP research collaboration among five clinical sites. NeMUP subjects were recruited from the Prevention Project Dunkelfeld (“Don’t offend”) for self-identified pedophiles seeking help, as well as prisons, online forums, advertisements, and mailing lists. P+CSO: M=39.8 (9.00), P-CSO: M=34.2 (9.40), NPC: M=33.6 (10.2)	MRI scan, the TFCE_mediation toolbox use for cortex-wise analyses of cortical surfaces and voxel-wise analyses of white matter FA, general cognitive ability was measured using the Wechsler Abbreviated Scale of Intelligence II	• Lower full-scale IQ (FSIQ) performance was strongly predict with P+CSO. • P+CSO had lower CT in the right motor cortex and pronounced reductions in SA spanning the bilateral frontal, temporal, cingulate, and insular regions, and lower FA particularly in the corpus callosum. • The relationship between SA and P+CSO was significantly mediated by FSIQ, particularly in the prefrontal and anterior insular cortices. • Within P+CSO, left prefrontal and right anterior cingulate SA negatively correlated with number of CSOs.
12	Massau et al. (2017b) (27)	Germany	To assess differences in the neural mechanisms of moral judgment associated with pedophilia and/or sexual offending against children, using functional imaging technique in combination with a new offense scenario evaluation task.	31 pedophilic men (16 P+CSO, 15 P-CSO) and 19 healthy controls. It is a single-site project of a multisite research collaboration on the neural mechanisms underlying pedophilia and sexual offending against children (NeMUP). 10 P+CSO's: those can be divided into ‘darkfield’ (n=4) offenders which refers to the fact that their former offense was not accused or sentenced by the German law system, those who already served a prison sentence for a child sexual offense (n=5) and one participant who had a pending proceeding up to the time of data acquisition. The other six pedophilic offenders were recruited from correctional services in the state of North Rhine-Westphalia, Germany. The HC group was recruited by advertisements in municipal institutions in city of Essen, Germany. All participants were age between 20–55 years, no current diagnosis of an axis I psychiatric disorder besides paraphilia, no neurological disorders, no mental retardation, explicit sexual orientation (either hetero- or homosexual), no contraindications to magnetic resonance imaging as well as no psychopharmacological treatment or other medication that affect sexual functioning. P+CSO: M=36.44 (8.01), P-CSO: M=31.73 (6.49), HC: M=33.47 (10.24)	Structured Clinical Interview (SCID) for DSM-IV-TR, estimation of global intelligence using the German version of the Wechsler Adult Intelligence Scale (4th Edition), sexual orientation and preference was then confirmed by means of the Kinsey scale for developmental stages, behavioral data using decision duration, reaction times (RT) and responses, fMRI.	• Scenarios depicting sexual offenses against children compared to those depicting adults, healthy controls showed higher pattern of activation in the left temporo-parietal-junction (TPJ) and left posterior insular cortex, the posterior cingulate gyrus as well as the precuneus, comparing to pedophilic men and vice versa. • Brain activation in these areas were positively associated with ratings of moral reprehensibility and negatively associated with decision durations, but only in controls. • Brain activation, found in key areas related to the broad network of moral judgment, theory of mind and (socio-) moral disgust - point to different moral processing of sexual offenses in pedophilia in general.
13	Ponseti et al. (2014) (28)	Germany	Using the blood-oxygen-level-dependent (BOLD) signal, performing a functional MRI to delineate the brain regions processing the sexually preferred age cues of faces (regional brain responses related to the sexual attractiveness).	Fifty-six men (11 heterosexual pedophiles, 13 homosexual pedophiles, 18 heterosexual teleiophiles and 14 homosexual teleiophiles) Mean age: NOT PROVIDED	International Affective Picture System (IAPS), fMRI, blood- oxygen-level-dependent (BOLD) signal as an index of regional brain activity	• Common pattern of brain activity covering the inferior occipital gyrus (IOG) (bilaterally), the fusiform gyrus (FFG) (bilaterally), the left nucleus caudatus (NC) (head and tail), the putamen, the sulcus calcarinus (bilaterally) and the left ventrolateral prefrontal cortex (VLPFC).
14	Ponseti et al. (2016) (29)	Germany	To classify participants as pedophilic or non- pedophilic solely on the base of their hemodynamic response to adult and child faces/applying a neurofunctional pattern classification to assess pedophilia.	24 men pedophiles, 32 men teleiophiles. Participants, procedure, stimuli and fMRI analysis were described in detail (30). Eleven of the pedophilic participants were part of the ‘Dunkelfeld’ prevention project (31). The remaining 13 pedophiles were treated in our outpatient department. Ten pedophiles had been sentenced earlier in life, with only three of them having served a prison sentence. Groups were matched for age and intelligence. Unclear control’s group. 11 heterosexual pedophiles: M=37 (5.9), 13 homosexual pedophiles: M=33.5 (14.2), 18 heterosexual teleiophiles: M=32.4 (8.2), 14 homosexual teleiophiles: M=28.6 (5.7)	DSM-IV-R, MRI, International Affective Picture System, BOLD	• Significant differences of the individual difference maps between pedophilic and teleiophilic participants in extended brain areas (girls vs. women and boys vs. men comparison).
15	Ponseti et al. (2018) (32)	Germany	To evaluate whether there are hints of an aberrant nurturing processing in pedophilic men.	150 male subjects participated in the main experiment; fMRI data was analyzed for 115 subjects. 17 pedophilic adults and 17 teleiophilic adults were raters of colored images of infant mammals and adult mammals of the same species. Raters did not participate in the fMRI measurements. 60 subjects met ICD-10 diagnostic criteria for pedophilia. Pedophilic subjects were recruited from four outpatient departments of forensic psychiatry or sexual medicine in Berlin, Hamburg, Hannover, and Kiel, and the community using the official NeMUP-website as well as by various German Internet forums to inform self-identified pedophilic men about the study. Pedophiles: M=36.6 (10.7), Teleiophiles: M=35 (10.2)	Intelligence using the German version of the Wechsler Adult Intelligence Scale (WAIS-IV), handedness by a handedness inventory, and sexual gender orientation. Behavioral pilot experiment: colored images of infant mammals and adult mammals of the same species were rated on a nine-point Likert-type scale in terms of sexual arousal, valence, and unspecific arousal. Main experiment: participants exposed to the animal images in a block design consisting pictures of infant and adult animals. fMRI	• Pedophilic raters and teleiophilic raters did not perceive the animal pictures as sexually arousing as indicated by the corresponding mean ratings. • Only in pedophiles did infants relative to adult animals increase brain activity in the anterior insula, supplementary motor cortex, and dorsolateral prefrontal areas. • Within-group analysis revealed an increased brain response to infant animals in the left anterior insular cortex of the pedophilic participants.
16	Popovic et al. (2023) (33)	Germany	To use machine learning and MRI data to identify PO individuals.	29 males from a single center: 14 pedophilic offenders & 15 healthy controls (age & education matched). Participants were inpatients at the Forensic Psychiatric State Hospital in Uchtspringe, Saxony-Anhalt, Germany. The external HC sample were scanned in the same MRI unit as part of a different study cohort. Patients: M=40.07 (8.76), Controls: M=44.13 (11.53)	MRI based on the eigenvalues, the mean diffusivity (MD) and fractional anisotropy (FA) as well as the axial diffusivity (AD) and radial diffusivity (RD) coefficients were determined. Linear support vector machine to discriminate between PO and HC individuals using these WM microstructure data.	• Higher fractional anisotropy in the left amygdala, the right dorsal anterior cingulate cortex, bilaterally in the pregenual anterior cingulate cortex and in the right rostral anterior cingulate cortex was strongly predictive of PO individuals. • Stronger structural connectivity values in the left hemisphere, were among the main predictors of PO individuals (left amygdala to left prefrontal cortex, left prefrontal cortex to left amygdala).
17	Ristow et al. (2018) (34)	Germany	To assess whether in the dorsal anterior cingulate cortex (dACC), a frontal brain region subserving attentional control of behavior and perception of salient stimuli, presumed dysfunctions in GABAergic metabolism linked to actual abusive behavior in pedophilic sex offenders. To examine whether GABAergic deficits in pedophilic patients with hands-on delinquency associated with behavioral response patterns including impulsivity and self-control, measured with German versions of the Barratt Impulsiveness Scale and the German ADHD Self-Report Scale.	10 pedophilic sex offenders and 10 matched controls for gender, group size, age and laterality index, within the framework of a German multi-site research project NeMUP. Pedophiles: M=33.70 (10.46), Healthy Controls: M=31.60 (4.27)	Barratt Impulsiveness Scale, German ADHD Self-Report Scale, German Hamilton Anxiety Rating Scale, German 21-item Hamilton Depression Scale, Tanner stages, German version of the Wechsler Adult Intelligence Scale, MRI, MRS-acquisition	• In dACC but not in the control region pedophilic sex offenders showed reduced GABA/Cr concentrations compared to healthy controls. • Reduced GABA/Cr in patients was correlated with lower self-control measured with the Barratt Impulsiveness Scale.
18	Schiffer et al. (2017) (35)	Germany	To differentiate brain structural anomalies in pedophiles with and without a history of CSO.	219 men were cross-sectionally recruited at four sites within the NeMUP at four acquisition sites in Germany: 58 pedophiles with a history of CSO, 60 pedophiles without any history of CSO and 101 non-pedophilic, non-offending controls. The participants were recruited via online advertisements, forum posts and email lists. The recruitment methods were the same for both pedophilic groups, which additionally but not exclusively comprised participants recruited from legal and clinical institutions, including the ‘Prevention Project Dunkelfeld’. P+CSO: M=40.1 (9.1), P-CSO: M=34.4 (9.2), HC: M=33.8 (10.5)	ICD-10, Kinsey scale, Tanner stages, MRI scanner, DSM-IV-TR, viewing reaction time (VRT), the Wechsler Adult Intelligence Scale, 4th Edition, the Barratt Impulsiveness Scale (BIS-11), the German short version of the Interpersonal Reactivity Index (SPF-IRI), the German version of the SIS/SES questionnaire, the Edinburgh Handedness Inventory, 2D:4D digit ratio, the Screening Scale for Pedophilic Interest, 2nd version (SSPI-2).	• No significant differences between the gray matter (GM) volumes of the non-offending pedophiles and the teleiophilic controls. • The pedophiles who had engaged in CSO showed a significantly reduced relative (GM) volume in the right temporal pole (TP) compared with pedophiles who did not. This difference was not attributable to age, level of education, IQ, sexual orientation, drug misuse/dependence, other Axis I or II disorders or general criminality. • The GM volume in the right TP was negatively associated with self-focused sexual behavior in both healthy controls and offending pedophiles and the viewing reaction time index, which is a measure of pedophilic tendencies in healthy controls. • The GM volume in the right TP was positively correlated with affective empathic skills and the propensity to inhibit sexual behavior due to potentially negative consequences of sex in non-offending pedophiles. • Regression analysis revealed that the lower GM volume of the dorsomedial prefrontal or anterior cingulate cortex was associated with a higher risk of re-offending in pedophilic child molesters.
19	Schuler et al., 2022 (36)	Germany	To explore the neural correlates of Cognitive Empathy (CE) in subjects with pedophilia with (P + CSO) and without (P− CSO) child sexual offending.	15 P + CSO, 15 P − CSO and 24 teleiophilic male controls (TC) performed a Cognitive Empathy task during fMRI. Participants were recruited within the Berlin site of the research network ‘NeMUP’ comprising five German collaborative research sites. Pedophilic men were additionally (not exclusively) recruited from practitioners and the “Prevention Project Dunkelfeld”, offering anonymous and confidential treatment to self-identified and undetected pedophilic individuals. P + CSO: M=41.0 (9.0), P - CSO: M=38.8 (10.5), TC: M=36.8 (12.9)	Three subscales [Perspective Taking, Personal Distress (PD) and Emotional Concern] of the Interpersonal Reactivity Index (IRI) were employed to assess self-reported empathic functioning. The cognitive empathy task consisted of 16 cartoon stories with 8 CE trials and 8 control trials. During the CE condition, participants were instructed to take the perspective of the story’s protagonist and judge via button press changes in his/her affective state (better, worse, equal compared to the preceding picture). Participants performed a CE task during fMRI.	• Reduced activation was observed in the left precuneus (Pcu) and increased activation in the left anterior cingulate cortex (ACC) in P − CSO compared to P + CSO. • There was also evidence for increased right superior temporal gyrus activation in P − CSO that might constitute a potentially compensatory recruitment due to the dampened Pcu activation. • Correlations: In P − CSO, higher levels of self-reported PD of the IRI were associated with greater left Pcu deactivation. Decreasing intelligence was associated with increasing superior temporal gyrus (STG) activity in P − CSO.
20	Storch et al. (2023) (37)	Germany	To accurately investigate the structure of the hypothalamus in pedophilia with or without CSO.	73 P+CSO, 73 P-CSO, 133 non-pedophilic, non-offending controls (all-male sample). Participants were recruited in the context of the multi-center study “Neural Mechanisms Underlying Pedophilia and Sexual Offending against Children” (NeMUP). Subjects were either participants of the “Prevention Project Dunkelfeld”, where self-identified people with pedophilia are offered anonymous therapy or they were recruited via prisons, online advertisements, forum posts, and mailing lists, or during fulfillment of a suspended sentence. P+CSO: M=39.99 (9.17), P-CSO: M=34.78 (9.35), Controls: M=33.35 (10.10)	ICD-10, DSM-IV-TR, modified version of the Kinsey Scale for developmental stages, Tanner stages I-V, German Edinburgh Handedness Inventory (EHI), German version of the third edition of the Wechsler Adult Intelligence Scale (WAIS), MRI.	• Men with pedophilia who committed CSO on average had a 47 mm^3^ smaller hypothalamus per side than people without committed CSO. • This effect was driven by both the group of non-offending people with pedophilia and the control group. • By contrast, the exploratory comparison of pedophilic persons without CSO with the control group showed no significant difference.
21	Szczypinski et al. (2022) (38)	Poland	To examine differences in brain function and behavior between CSO+ and CSO- patients regarding emotional interference on cognitive processes and inhibition.	11 CSO+ and 14 CSO- male patients as well as 17 matched healthy controls. CSO+ and CSO- participants were diagnosed with PD based on the DSM-V and the ICD-10 criteria. Both CSO groups were recruited from the Department of Sexology of the Nowowiejski Hospital in Warsaw. The HC group was recruited through advertisements on social media and matched according to education years. CSO+: M=43.8 (8.46), CSO-: M=36.56 (8.8), HC: M=32.24 (7.85)	An affective Go/NoGo task in a block design appropriate for a functional magnetic resonance imaging (fMRI) experiment. This task comprised the following conditions: Negative Go, Negative NoGo and two corresponding conditions with neutral pictures (Neutral Go and Neutral NoGo). Each block started with a cue showing the block type (Go or NoGo) and comprised 14 trials. fMRI data analysis was to the dorsolateral prefrontal (DLPFC), orbitofrontal, and anterior cingulate cortices and the general linear approach in SPM12 software was used.	• The HC and CSO- groups, but not the CSO+ group, showed significantly slower reactions in negative blocks compared with neutral blocks. • Brain analysis revealed increased activation in the right dorsolateral prefrontal cortex during emotional interference contrast (Negative > Neutral) in the HC and CSO- groups; however, there was decreased activation in the CSO+ group. • In the CSO+ group, negative distractors did not increase cognitive control processes, which was observed in the CSO- and HC groups at the behavioral and neural levels.
22	Weidacker et al. (2022) (39)	Germany	Focused on interference inhibition and examined event-related functional magnetic resonance imaging (fMRI) data of three groups of men performing a color-word Stroop task: (1) pedophiles with a history of CSO, (2) pedophiles without a history of CSO and (3) non-pedophilic, non-offending healthy controls.	11 pedophiles with a history of CSO (P+CSO, n = 11), 8 pedophiles without a history of CSO (P–CSO, n = 8) and 10 non-pedophilic, non-offending healthy controls (HC, n = 10). Pedophilic participants were recruited via explicit online forums and the research groups’ website as well as via the ‘Prevention Project Dunkelfeld’ and from correctional services in North Rhine-Westphalia, Germany (NeMUP). Non-pedophilic controls were recruited from the community via flyers and advertisements in public institutions. P+CSO: M=43.55 (11.58), P-CSO: M=33.25 (10.79), HC: M=37.70 (13.12)	Event-related functional magnetic resonance imaging (fMRI) performing a color-word Stroop task, presenting words in either congruent or incongruent color against a black background.	• On the behavioral level, P+CSO revealed increased Stroop interference as compared to P–CSO and HC. • Increased Stroop interference in P+CSO was accompanied by enhanced conflict-related activity in left superior parietal cortex and precentral gyrus as compared to P–CSO. • P–CSO showed increased post-error-related activity in left posterior cingulate, precuneus and middle temporal gyrus as compared to P+CSO.

**Table 2:** Study characteristics and main findings on genetic/epigenetic and biochemical biomarkers.

Genetic/Epigenetic and Neuroendocrinal biomarkers						
No	Authors, year	Origin of study	Aim/Outcome	Sample type, size and mean age (SD)	Testing methods	Findings
1	Cazala et al. (2019) (19)	France	To identify through functional magnetic resonance imaging the brain responses of patients with pedophilic disorder to visual stimuli depicting children and compare them with matched healthy controls.	25 male outpatients with pedophilic disorder, 24 male healthy controls matched on sexual orientation (to female or male adults), handedness, age (range 18-65) (Patients: M=42.2 (12.0), Controls: M=37.8 (12.8)	Handedness using Edinburgh Handedness Inventory, IQ using Wechsler Adult Intelligence Scale-III, Sexual function using Sex History Questionnaire Revised (SHQ-R), the Sexual Fantasies, Desires and Activity Interview Schedule (SFDAIS), the Sexual Interest Score (SIS), the Brief Sexual Function Questionnaire (BSFQ), the Sexual Arousal Inventory (SAI), hormonal investigation (blood sample for testosterone, sex-hormone binding globulin SHBG, LH, FSH, prolactin, and cortisol), fMRI using different categories of pictures (stimuli), penile plethysmography, behavioral and endocrine data	• Significantly lower mean testosterone levels in pedophilic patients compared to control group.
2	Fonteille et al. (2019) (20)	France	To identify through positron emission tomography the brain responses of males with pedophilic disorder to validated visual sexual stimuli depicting children (VSSc) and to compare them with male healthy controls.	15 male outpatients with pedophilic disorder, 15 male healthy controls matched for sexual orientation, age, and handedness. Patients: M=42.0 (12.6), Controls: M=41.2 (16.2)	DSM-IV-TR, ICD-10, the Edinburgh Handedness Inventory score, of the Wechsler Adult Intelligence Scale-III, the Sex History Questionnaire Revised SHQ-R, questionnaire on Sexual Fantasies, Desires and Activity (SFDAQ), the Brief Sexual Function Questionnaire (BSFQ), the Sexual Arousal Inventory (SAI), the Symptom Checklist-90-Revised (SCL-90R), the 21-item Beck Depression Inventory version, the Barratt Impulsiveness Scale (BIS-10), sex-hormone binding globulin (SHBG), bioavailable testosterone (not bound to SHBG), estradiol, LH, FSH, prolactin, cortisol, PET scan, volumetric penile plethysmograph	• Lower plasma levels of total testosterone and of bioavailable testosterone, (unbound to the Sex-Hormone Binding Globulin), were in patients.
3	Jahn et al. (2022) (40)	Germany	To investigate the methylation rates of HTR3A and the serotonin transporter SLC6A4 as well as the SLC6A4LPR as a potential contributing factor to pedophilic preference and/or sexually disinhibited behavior.	Blood samples from a total of 261 study participants: 60 subjects with a pedophilic sexual preference with CSO (P + CSO); 59 subjects with a pedophilic sexual preference without CSO (P–CSO); 25 substitute offenders (CSO–P), who were imprisoned due to committed sexual child abuse without pedophile tendency and 117 healthy controls (HC). consortium (NeMUP) study P+CSO (n = 83): M=40.02 (9.55), P-CSO (n = 79): M=34.65 (9.67), CSO-P (n = 32): M=43.31 (12.78), HC (n = 148): M=33.64 (9.96)	DNA from blood samples was extracted with the automated Nucleomag DNA from blood-Kit (Macherey Nagel®). Analyses of methylation rates by epigenetic sequencing methylation analysis (ESME). SLC6A4LPR genotyping.	• Methylation rates of HTR3A showed significant differences between child sexual offenders and non-offenders, with child sexual offenders showing lower methylation rates. • HTR3A methylation rates showed significant negative correlations • with the Child Trauma Questionnaire (CTQ) subscale “sexual violence”, and the number of sexual offenses committed. • For HTR3A, significant higher methylation rates were detected in pedophilia compared to non-pedophilic controls, whereas for SLC6A4 methylation rates were reduced.
4	Jakubczyk et al. (2017) (41)	Poland	To identify an association between a history of sexual offense and the distribution of genotypes and alleles in the analyzed polymorphisms.	97 cases (65 pedophilic child molesters and 32 rapists) and 76 controls, all men, matched by sex and age to the study group. Paraphilic Sexual Offenders: M=39.4 (11.2), Controls: M=36.1 (13.3)	Mini-Mental State Examination, Mini-International Neuropsychiatric Interview, Saliva for genes associated with DA activity: dopamine receptor genes (DRD1, DRD2, DRD4), catechol-O-methyltransferase gene (COMT), dopamine transporter gene (DAT), genes associated with 5-HT activity: serotonin transporter gene (SLC6A4), serotonin type 2A receptor gene (5HTR2A), tryptophan hydroxylase 2 gene (TPH2), monoamine oxidase A gene (MAOA), and genes associated with brain development: brain-derived neurotrophic factor gene (BDNF).	• No association between a history of sexual offense and the analyzed polymorphisms associated with DA and 5-HT activity.
5	Kruger et al. (2019) (42)	Germany	To investigate prenatal, genetic, and epigenetic parameters of the androgen system in a sample of child sexual offenders with and without pedophilia, non-offending pedophiles, and controls.	194 subjects: 57 P+CSO, 45 P−CSO, 20 CSO−P, (German Prevention Project Dunkelfel) and 72 controls matched for age and intelligence. The sample was part of the NeMUP study (multicenter study in Germany dedicated to disentangling the clinical, neuropsychological, and neurobiological underpinnings of pedophilia and CSO). P+CSO: M=39.5 (8.7), P-CSO: M=36.8 (9.1), −P+CSO: M=40.5 (11.8), −P−CSO:M=38.2 (9.9)	Finger length ratio 2D:4D, endocrine analyses and DNA extraction by blood samples	• CSOs showed signs of elevated prenatal androgen exposure compared with non-offending pedophiles and controls. • The methylation status of the androgen receptor gene was higher in CSOs, indicating lower functionality of the testosterone system, with lower peripheral testosterone levels. • Interaction effect on methylation levels between offense status and androgen receptor functionality. • Negative association between markers of prenatal androgenization and number of child sexual offenses, and positive association between methylation level and total number of child sexual offenses. • Marginally lower testosterone/cortisol ratio in child sexual offenders compared with non-offending subjects.

**Table 3:** Study characteristics and main findings on physiological biomarkers.

Physiological biomarkers						
No	Authors, year	Origin of study	Aim/Outcome	Sample type, size and mean age (SD)	Testing methods	Findings
1	Cazala et al. (2019) (19)	France	To identify through functional magnetic resonance imaging the brain responses of patients with pedophilic disorder to visual stimuli depicting children (VSc) and to compare them with matched healthy controls.	25 male outpatients with pedophilic disorder, 24 male healthy controls matched on sexual orientation (to female or male adults), age (18-65), and handedness were recruited through a web platform Patients: M=42.2 (12.0), Controls: M=37.8 (12.8)	Handedness using Edinburgh Handedness Inventory, IQ using Wechsler Adult Intelligence Scale-III, Sexual function using Sex History Questionnaire Revised (SHQ-R), the Sexual Fantasies, Desires and Activity Interview Schedule (SFDAIS), the Sexual Interest Score (SIS), the Brief Sexual Function Questionnaire (BSFQ), the Sexual Arousal Inventory (SAI), blood sample (testosterone, sex-hormone binding globulin SHBG, LH, FSH, prolactin, and cortisol), fMRI using different categories of pictures (stimuli), plethysmography	Plethysmography results • *Contrast 1* children of the patients’ preferred gender: not significant • *Contrast 2* pictures of adults: higher responses to pictures of undressed adults, i.e. only condition effect was significant • *Contrast 3* pictures of characters with both the age and the gender preferred by each group: as results as in Contrast 2, i.e., only the condition effect was significant. • *Contrast 4* Patients showed higher penile responses to pictures of undressed children than to pictures of undressed adults. *Contrast 4b:* Including the Dressed Condition into the contrast [(ChPrefresponse)patients−(AdPrefresponse)patients], the Condition by Age of Character interaction was not significant. Higher responses to pictures of undressed characters and an Age of Character effect, with higher responses to pictures of children than to pictures of adults. • *Contrast 5* responses to adults’ versus children’s nudity: higher responses to pictures of children than to pictures of adults in patients and the reverse pattern in controls • *Contrast 6* comparison of patients who had committed a sexual offense with non-offenders on their responses to pictures of preferred children: no significant differences.
2	Dyshniku et al. (2015) (43)	Canada	To assess the prevalence of minor physical anomalies in a clinical sample of men	Two hundred and six (n=206) participants. 24 pedophiles, 55 hebephiles, and 52 teleiophiles, based on their phallometric response. Participants: M=37.2 (12.6)	forensic and medical file review, a semi-structured interview spanning offense and sexual history, a phallometric test for erotic preference, and a brief neuropsychological interview and battery. Waldrop Scale, Penile plethysmography (phallometry), Edinburgh Handedness Inventory	• Significant associations emerged between minor physical anomalies (MPA) indices and indicators of pedophilia, including penile responses to depictions of children, number of child victims, and possession of child pornography. • Greater sexual attraction to children was associated with an elevated craniofacial-to-peripheral anomalies ratio. • The craniofacial-to-peripheral ratio was composed of an increased number of craniofacial anomalies and a fewer than typical number of peripheral anomalies.
3	Fazio et al. (2014) (44)	Canada	To establish the pattern of atypical handedness in pedophilia.	All study participants (male) were recruited from the Kurt Freund Laboratory of the Centre for Addiction and Mental Health (Toronto, Ontario, Canada). Available for analysis were data from 1857 individuals assessed between the years 2000 and 2011. 1712 men to be classified as pedophilic (n=219), hebephilic (n=619), or teleiophilic (n=874). M=38.8 (13.6)	Edinburgh Handedness Inventory, volumetric phallometry, Phallometric Pedophilia Index (PPI)	• Pedophiles demonstrated high rates of non-right handedness, primarily manifested as sinistrality. • Those who had a sexual preference for pubescent children evidenced increased ambiguous handedness. • Analysis of laterality quotient (LQ) scores indicative of ambiguous-handedness demonstrated that this was the correct classification as opposed to mixed-handedness.
4	Fazio et al. (2017) (45)	Canada	To investigate the relationship between measured height and pedophilia	102 cases (20 Pedophiles, 36 Hebephiles, 46 Teleiophiles.) Participants were recruited from the Kurt Freund Laboratory of the Centre for Addiction and Mental Health (Toronto, Ontario, Canada). All participants: M=37.02 (12.03)	Assessment of minor physical anomalies, Torso length, Leg length, Phallometric assessment (penile plethysmography)	• Pedophiles demonstrated significantly shorter leg length than teleiophiles. • Torso length did not differ significantly between pedophiles and teleiophiles.
5	Fonteille et al. (2019) (20)	France	To identify through positron emission tomography the brain responses of males with pedophilic disorder to validated visual sexual stimuli depicting children (VSSc) and to compare them with male healthy controls	15 male outpatients with pedophilic disorder, 15 male healthy controls matched for sexual orientation, age, and handedness. Patients: M=42.0 (12.6), Controls: M=41.2 (16.2)	DSM-IV-TR, ICD-10, the Edinburgh Handedness Inventory score, of the Wechsler Adult Intelligence Scale-III, the Sex History Questionnaire Revised SHQ-R, questionnaire on Sexual Fantasies, Desires and Activity (SFDAQ), the Brief Sexual Function Questionnaire (BSFQ), the Sexual Arousal Inventory (SAI), the Symptom Checklist-90-Revised (SCL-90R), the 21-item Beck Depression Inventory version, the Barratt Impulsiveness Scale (BIS-10), sex-hormone binding globulin (SHBG), bioavailable testosterone (not bound to SHBG), estradiol, LH, FSH, prolactin, cortisol, PET scan, volumetric penile plethysmograph	• Compared with controls, patients showed a higher difference between Desire for Sexual Arousal (DSA) ratings for undressed and for normally dressed children. The same Group by Condition interaction was found for erectile responses to VSSc. • In response to VSSc, the between-groups analysis showed that activation in the right inferior temporal cortex [Brodmann area (BA) 20] was lower in patients than in controls. • In patients but not in controls, the presentation of VSSc induced an activation in a more caudal region of the right inferior temporal gyrus (BA 37) and in the left middle occipital gyrus (BA 19). • In patients the level of activation in the caudal right inferior temporal gyrus was positively correlated with ratings of sexual arousal elicited by VSSc, whereas this correlation was negative in BA 20.
6	Fromberger et al. (2013) (46)	Germany	Test hypotheses 1. pedophiles show shorter entry time to child stimuli than to adult stimuli, and that non-pedophiles show shorter entry time to adult stimuli than to child stimuli. 2. pedophiles demonstrate a longer relative fixation time to child stimuli than to adult stimuli, and that non-pedophiles demonstrate longer relative fixation time to adult stimuli than to child stimuli.	22 pedophiles, 8 non-pedophilic forensic controls/rapists (forensic inpatients) with no sexual assault upon children in their history, and 52 healthy controls. Pedophiles: M=42.06 (4.50), Forensic controls: M=36.88 (5.09), Non-forensic controls: M=25.27 (1.01)	ICD-10, German version of the Wechsler Adult Intelligence Scale (WAIS), SCID-II, DSM-IV, the Kinsey Scale, Screening Scale for Pedophilic Interests (SSPI), SMI iView XTM RED eye tracker, an iView XTM workstation, BeGaze 3 (Senso-Motoric Instruments GmbH)	• Pedophiles demonstrated significantly shorter entry time to child stimuli than to adult stimuli. • The opposite was the case for non-pedophiles, as they showed longer relative fixation time for adult stimuli, and, against expectations, pedophiles also demonstrated longer relative fixation time for adult stimuli.
7	Jordan et al. (2016) (47)	Germany	To analyze attentional control processes in a sexual distractor task, in pedophiles, forensic control patients, and healthy subjects.	22 male pedophilic subjects, 7 male forensic inpatients without any history of sexual assault against children, 50 male healthy subjects. Pedophiles: M=42.09 (10.92), Forensic controls: M=34.86 (14.28), Non-forensic controls: M=25.38 (7.39)	ICD-10, Kinsey scale, the Screening Scale for Pedophilic Interests, Wechsler Adult Intelligence Scale (German version), SMI iView X RED eye tracker	Pedophiles demonstrated significantly lower attentional control in the sexual distractor task than both control groups (non-pedophiles). • They showed a shorter fixation latency and longer fixation time for sexual distractors and a longer fixation latency and shorter fixation time for cognitive target stimuli compared to non-pedophiles. • • For classification analyses, an attentional control index (ACI) (the difference between • eye movements on cognitive target stimuli and sexual distractors) was created. For fixation latency, a good classification between pedophiles and non-pedophiles was found.
8	Jordan et al. (2018) (48)	Germany	To compare outpatients with a self-reported sexual interest in children to pedophilic forensic inpatients and non-pedophilic controls with respect to sexual interest and attentional control, both measured independently of self-reports.	11 outpatient men with self-reported sexual interest in children, 22 pedophilic forensic inpatients, 60 male non-pedophilic control group. The outpatient preventive treatment project “PsM”. Information for forensic inpatients was based on file records. Outpatient group had been diagnosed and treated in the PsM-project. Data from this control group and the forensic inpatient group come from earlier research projects (Fromberger et al., 2012b (46); Jordan et al.,2016b (47)). Outpatients: M=39.18 (8.77), Inpatients: M=42.09 (10.92), Controls: M=26.82 (9.28)	ICD-10, Not-Real-People (NRP) picture set, SMI iView X RED eye tracker	• Outpatients showed similar sexual interest in children as pedophilic forensic inpatients, and demonstrated significantly better attentional control than pedophilic forensic inpatients in the face of adult sexual stimuli, but the difference regarding child sexual stimuli did not reach significance. • Outpatients with a self-reported sexual interest in children exhibit a good attention control in the face of adult and cognitive stimuli.
9	Knott et al. (2016) (49)	Canada	To investigate the time course of erotic picture processing in patients with pedophilia using event-related brain potentials (ERPs).	22 male pedophilic patients and 20 male healthy controls matched for age, handedness and educational level. All patients were “admitters”, exclusively attracted to female children, and were participants in the Sexual Behaviours Clinic (SBC) within the Integrated Forensics Program of the Royal Ottawa Mental Health Centre (ROMHC). The comparison group was recruited by local media and internet advertisements. Patients: M=43.82 (2.61), Controls: M=41.55 (2.75)	Structural Clinical Interview (SCID), DSM-IV, self-rated Kinsey Scale, the Bumby Molest Scale (BMS) II, International Affective Pictures System (IAPS), EEG	• Early latency anterior ERP components were highly selective for erotic pictures. • The ERPs elicited by emotional stimuli were similar in patients and controls, but an early frontal positive (P2) component was significantly attenuated and slow to onset in pedophilia, and correlated with a clinical measure of cognitive distortions.
10	Rosburg et al. (2018) (50)	Switzerland	To investigate response inhibition and error processing of pedophilic CSOs in a Go/Nogo-task by event-related brain potentials (ERPs) to reveal whether these offenders exhibit, aside from behavioral deficits, neurocognitive alterations in the processing of task cues and commission errors.	21 contact CSOs, 19 non-contact CSOs, 21 controls (part of the Basel Measurable Indicators of Pedophilic Sex Offenders MIPS study). Controls were recruited by advertisements in newspapers and negated any sexual interest in prepubescent children. P+CSO: M=35.3 (10.9), Non-contact CSO: M=37.8 (9.7), Controls: M=30.8 (10.2)	Go/Nogo task, DSM-IV-TR, Edinburgh Handedness Inventory, Alcohol Use Disorder Identification Test (AUDIT),	• CSOs showed a slight, but non-significant increase of the false alarm rate to Nogo cues, as compared to controls. • The amplitudes of the ERP components N2 and P3 to Nogo cues followed by correctly withhold responses did not vary between CSOs and controls. • The analysis of the ERPs to committed errors showed that the Ne amplitudes (reflecting error detection) did not differ between the groups either, whereas the Pe amplitudes (reflecting error evaluation and error awareness) were strongly diminished in CSOs. • This diminishment was primarily found in contact CSOs.

**Table 4:** Study characteristics and main findings on cognitive/behavioral biomarkers.

Cognitive/Behavioral biomarkers						
No	Authors, year	Origin of study	Aim/Outcome	Sample type, size and mean age (SD)	Testing methods	Findings
1	Abé et al. (2021) (16)	Sweden	To identify clinically useful biomarkers and risk factors by investigating PD-related neurobiology using magnetic resonance imaging (MRI), psychiatric assessment, and cognitive testing.	55 self-referred, help-seeking, non-forensic male patients with DSM-5 PD were recruited through PrevenTell, a Swedish national helpline, 57 age-matched, healthy male controls (HC) screen-negative for PD, were recruited in the Stockholm catchment area through Karolinska Trial Alliance and adverts on Karolinska Institute's homepage. PD: M=36 (12), HC: M=36 (12)	Psychiatric Comorbidity & Clinical Symptoms: M.I.N.I. 6.0.0 for coexisting psychiatric disorders, RAADS-14 for autism spectrum disorder, ASRS-v1.1 for ADHD symptoms. General cognitive ability: WAIS-IV. Anthropometric measurements: participants’ height, weight, lengths of index finger (2nd digit) and ring finger (4th digit) of the right hand for assessment of the 2D:4D digit ratio. Structural MRI image acquisition and processing	• PD patients had lower total IQ than HC.
2	Azizian et al. (2016) (51)	USA	To detect cognitive differences between individuals with a paraphilia diagnosis when compared with matched controls.	114 individuals with pedophilia and 56 individuals with paraphilia not otherwise specified (NOS), nonconsent, diagnoses, who were detained or civilly committed to a forensic psychiatric hospital. Age-, education-, and ethnicity-matched controls (n =99) were drawn from the RBANS normative standardization sample. The study utilized a 6-year (2005 to 2011) retrospective review of the clinical records of individuals that were detained or civilly committed to a state forensic psychiatric hospital pursuant to the California SVP Act. Pedophilia: M=50.66 (10.69), Paraphilia NOS: M=49.84 (7.88), Matched Controls: M=53.1 (17.97)	Repeatable Battery for the Assessment of Neuropsychological Status (RBANS), Wechsler Abbreviated Scale of Intelligence (WASI), and Wide Range Achievement Test 4 (WRAT4), Diagnostic and Statistical Manual of Mental Disorders (4th ed., text rev.; DSM-IV-TR)	• Individuals diagnosed with pedophilia and paraphilia NOS, nonconsent, obtained lower scores than matched controls based on the RBANS Immediate Memory, Visuospatial/Constructional, Delayed Memory indices and Total Score. • In comparison with individuals with paraphilia NOS, nonconsent, those with pedophilia diagnosis had lower scores on the RBANS Delayed Memory.
3	Franke et al. (2019) (52)	Germany	To (1) assess response inhibition, attention, and problem solving in convicted pedophilic child sexual offenders, and (2) compare the results with an IQ-matched sample of non-offending psychiatric patients and the norms for the tests.	15 male forensic inpatients (FIP) from 2 forensic inpatient mental health services, Guenzburg and Kaufbeuren, in Bavaria, Germany, and 15 IQ-matched control group were men living in 3 assisted living facilities for mentally disordered individuals associated with general mental health care institutions in the same districts of Bavaria. FIP: M=50.5 (11.4), CG: M=48.1 (11.0)	Neuropsychological assessment included subtests Go/NoGo, Alertness, and Divided Attention from the Test of Attentional Performance (TAP) and the German version of the Tower of London test (TL-D)	• No significant differences in the neuropsychological profiles of the two groups. • The FIP showed a higher rate of errors in the Go/NoGo test, indicating speed-accuracy trade-offs, i.e., the participants reacted just as fast as the standard population, but their high speed resulted in more mistakes.
4	Gibbels et al. (2019) (22)	Germany	To analyze clinical, neuropsychological and neurobiological features of convicted and non-convicted CSOs to unravel possible factors that might increase or decrease the probability of being convicted.	31 non-convicted men CSOs and 43 men convicted CSOs. Participants were recruited as part of the NeMUP study. Some subjects were recruited in prisons or during fulfillment of a suspended sentence. The analysis of the Go/No-go paradigm combined with fMRI included 23 non-convicted CSOs and 38 CSOs (not all participants fulfilled the criteria to be examined with MRI). Non-Convicted CSOs (n = 31): M=40.4 (10.8), Convicted CSOs (n = 43): M=39.8 (9.0)	MRI, DSM structured clinical interview (SCID), intelligence using WAIS-IV, empathy using multifaceted empathy test (MET), impulsivity Barratt impulsiveness scale (BIS-11), Go/Nogo task	• Non-convicted CSOs were neither more intelligent nor less impulsive nor suffered less from psychiatric disorders than convicted CSOs in this study. • No differences could be found concerning their age, the number of victims or the level of empathy. • Interestingly, convicted CSOs committed more delicts than non-convicted CSOs. • Non-convicted and convicted CSOs differed in sexual age orientation. There was a marginal significance concerning the number of male victims and the age of female victims.
5	Habermeyer et al. (2013) (23)	Switzerland	To assess how inhibition in pedophilia is related to a differential recruitment of frontal brain areas (functional imaging).	18 male right-handed subjects: 11 pedophilic subjects, 7 non-pedophilic controls. Pedophilic subjects (n = 11) were recruited from an outpatient cognitive behavioral group therapy at the Forensic Psychiatric Hospital, Basel, Switzerland. Seven control subjects were recruited using an advert on the University Hospital bulletin board. Pedophiles: M=49 (12.5), Controls: M=47 (8.6)	Go/NoGo Task, MRI scanner, Multiphasic Sex Inventory, revised German version of the Wechsler Adult Intelligence Scale	• Pedophilic subjects showed a slower reaction time and less accurate visual target discrimination.
6	Kargel et al. (2017) (25)	Germany	To analyze inhibitory control capacity and underlying inhibition-related neural activation pattern with respect to pedophilia and/or sexual offending against children, using event related fMRI in combination with a behavioral go/nogo paradigm.	117 men matched for age and IQ: 40 P+CSO, 37 P-CSO, 40 HC. Part of the (NeMUP) “Neural Mechanisms Underlying Pedophilia and Sexual Offending Against Children” project. P-CSO were recruited from the community (n=20) or the “Prevention Project Dunkelfeld” (PPD) (n=17). P+CSO were taken from the community (n=16), and the PPD (n=9), and from correctional institutions (n=15). Healthy controls recruited from the community throughout advertisements in public institutions included 40 men without a history of criminal behavior or current psychiatric disorders. P+CSO: M=38.25 (8.54), P-CSO: M=37.00 (8.84), HC: M=36.65 (10.13)	Go/No-Go paradigm, the Structured Clinical Interview for the DSM-IV-TR (SCID), modified version of the Kinsey scale for developmental stages, the German version of the Wechsler Adult Intelligence Scale, 4th Edition (WAIS-IV), MRI scanners.	• Compared to offending pedophiles, non-offending pedophiles exhibited superior inhibitory control as reflected by significantly lower rate of commission errors.
7	Lett et al. (2018) (26)	Germany	(1) To assess structural brain morphology using more recent MRI-based analysis approaches such as whole brain analyses of cortical thickness (CT), surface area (SA), and white matter fractional anisotropy (FA), (2) to differentiate between P+CSO, pedophiles who have not committed CSO (P-CSO) and non-pedophilic controls (NPC) in a large neuroimaging sample, and (3) to examine a potential neurobiological mechanism in which intelligence may mediate the association between aberrant cortical morphology and CSO behavior.	73 P+CSO, 77 P-CSO, 133 non-pedophilic controls (NPC). Male participants were recruited within the NeMUP research collaboration among five clinical sites. NeMUP subjects were recruited from the Prevention Project Dunkelfeld (“Don’t offend”) for self-identified pedophiles seeking help, as well as prisons, online forums, advertisements, and mailing lists. P+CSO: M=39.8 (9.00), P-CSO: M=34.2 (9.40), NPC: M=33.6 (10.2)	MRI scan, the TFCE mediation toolbox use for cortex-wise analyses of cortical surfaces and voxel-wise analyses of white matter FA, general cognitive ability was measured using the Wechsler Abbreviated Scale of Intelligence II	• Lower full-scale IQ (FSIQ) performance was strongly predict with P+CSO.
8	Massau et al. (2017a) (53)	Germany	To separate the influence of pedophilic preference and sexual offending against children regarding executive (dys-)function.	4 matched groups of men: (1) 45 P+CSO, (2) 45 P-CSO, (3) 19 CSO-P, and (4) 49 HC, German multi-sided research network NeMUP. The P-CSO group was composed of community dwelling pedophiles recruited via relevant Internet platforms or the German Prevention Project “Dunkelfeld”. Participants were matched regarding age, intelligence, handedness, and sexual gender orientation. The HC group was recruited from the community through advertisements in municipal institutions. P+CSO: M=38.04 (8.62), P-CSO: M=36.51 (9.46), CSO-P: M=40.26 (12.71), HC: M=36.43 (6.70)	Structured Clinical Interview (SCID) for DSM-IV-TR, General cognitive functioning using the German version of the Weschler Adult Intelligence Scale, 4th Edition, Sexual age and gender preference was confirmed by the Kinsey scale for developmental stages, Executive functioning using the Cambridge Neuropsychological Test Automated Battery (CANTAB): Stop Signal Task (SST), the Information Sampling Task (IST), Intra/Extradimensional Set Shift Task (IED), Spatial Working Memory Task (SWM), Stockings of Cambridge Task (SOC).	• Both CSO groups as compared to both non-CSO groups exhibited worsened response inhibition ability. • P+CSO performed best regarding cognitive flexibility. • Only non-pedophilic offenders showed additionally less ability for strategic working memory usage. • The non-pedophilic offender group was also characterized by worsened strategy usage in the Spatial Working Memory task as compared to healthy controls with both pedophilic groups performing in between. • The P+CSO group showed the best performance in set-shifting abilities • Performances were affected by age: only in pedophiles, response inhibition worsened with age, while age-related deficits in set-shifting abilities were restricted to non-pedophilic participants.
9	Picard et al. (2024) (54)	USA	To examine neuropsychological functioning in men with pedophilic disorder (PD), to assess whether findings from prior neuropsychological studies are replicated in a diverse sample including men with non-contact sexual offenses.	58 men who were convicted of a sexual offense, Patient Group (Pedophilic disorder): child sexual abuse material (CSAM) offenses (n=20), Control Group (No pedophilic disorder): 38 (men with contact sexual offenses n=33, non-contact sexual offenses n=5). Participants were recruited from two outpatient clinics in the northeast United States that specialize in the treatment of sexual offending behavior. Patients: M=46.95 (13.86), Controls: M=44.50 (11.61)	Intellectual Functioning: The Wechsler Adult Intelligence Scale-Fourth Edition (WAIS-IV) to estimate Full Scale IQ (FSIQ). Executive Functioning: the Controlled Oral Word Association Test (COWAT), the Stroop Color Word Test (Stroop), and the Trail Making Test (TMT). Processing Speed: the Symbol Search and Coding subtests of the WAIS-IV, as well as Trails A of the TMT. Attention: Digits Forward from the Digit Span subtest of the WAIS-IV and the first trial of the CVLT-II. Working Memory: the Digit Span and Arithmetic subtests of the WAIS-IV. Learning and Memory: The Brief Visuomotor Memory Test-Revised (BVMT-R) and the California Verbal Learning Test-Second edition (CVLT-II). Verbal Ability: the Vocabulary and Similarities subtests of the WAIS-IV, as well as Category Fluency. Visuospatial Ability: the Matrix Reasoning and Block Design subtests of the WAIS-IV. Effort: the Test of Memory Malingering (TOMM), as well as Reliable Digit Span index of the WAIS-IV (RDS), and the CVLT-II forced choice subscale.	• Participants with PD performed significantly better on verbal memory and visual discrimination than those without PD. • Men with PD made more errors on a set-shifting task but no significant differences were seen in domains of attention, intellectual functioning, visual learning and memory, visuospatial ability, or language ability.
10	Rosburg et al. (2021) (55)	Switzerland & Germany	To identify the potential of both indirect measures of sexual interest and general neuropsychological parameters to distinguish pedophilic CSOs from non-offending controls, as well as contact CSOs from noncontact CSOs.	21 contact CSOs (convicted of or had admitted to a contact sexual offense against a child), 20 non-contact CSOs (convicted of or admitted accessing, storing, or producing sexual material depicting children), 21 non-offending controls (CTLs) were recruited by advertisements in two local newspapers. CSOs were recruited among outpatients and inpatients of forensic-psychiatric hospitals in Switzerland and Germany. Participants were male gender, between 18 and 55 years of age, an IQ of 70 or above. Contact CSO: M=35.50 (10.64), Non-Contact CSO: M=37.19 (9.79), CTL: M=30.76 (10.15)	Cognitive functions: Fluid intelligence (IQ), Crystallized intelligence (IQ_C_), Alerting, Orienting, Risk taking, Resistance to interference, Episodic memory, Working memory errors. Indirect tests: IAT, VT, Semantic Misattribution Procedure (SMP), CRT task.	• CSOs committed significantly more working memory errors than CTL. • For the differentiation of CSOs from CTLs, indirect test parameters, such as slower responses in the CRT, longer VTs, or lower IAT scores, suggest the presence of pedophilic interest. • The profile of contact and noncontact CSOs was similar, with slightly increased levels of risk-taking behavior and greater susceptibility to interference in contact CSOs than in noncontact CSOs, that might be more related to the tendency to commit child sexual offenses.
11	Suchy et al. (2014) (56)	USA	To examine speed of information processing in three domains (motor speed, visual-perceptual speed, and visual-motor integration) among pedophilic child molesters (PEDs), non-pedophilic child molesters (N-PEDs), and non-sex offender controls (NSOs), and compare group data to national norms.	20 phallometrically identified PEDs, 20 N-PEDs, and 20 NSOs (all males). All participants resided in residential programs as an interim between incarceration and community reintegration. PED: M=34.15 (7.60), N-PED: M=31.90 (7.79, NSO: M=29.70 (6.60)	PPG, WAIS-III for visual-perceptual speed and the speed of visual-motor integration, Halstead-Reitan norms for motor speed, DSM–IV–TR, Chapman Handedness questionnaire, 25-item Wender Utah Rating Scale (WURS), Finger Tapping test from the Halstead-Reitan Battery, Inspection Time Task (ITT), Shipley Institute of Living Scales (SILS), Spatial Span from the Wechsler Memory Scale, Third Edition (WMS-III)	• PEDs exhibited slower visual perception and visual-motor integration than the other groups, with no differences for simple motor speed. • PEDs performed less accurately on the Inspection Time Task than the other groups, indicating that slow processing speed cannot be explained by a deliberate response style. • Group differences persisted after controlling for other potential confounds (age, estimate IQ, working memory, ethnicity, and substance use).
12	Szczypinski et al. (2022) (38)	Poland	To examine differences in brain function and behavior between CSO+ and CSO- patients regarding emotional interference on cognitive processes and inhibition.	11 CSO+ and 14 CSO- male patients as well as 17 matched healthy controls. CSO+ and CSO- participants were diagnosed with PD based on the DSM-V and the ICD-10 criteria. Both CSO groups were recruited from the Department of Sexology of the Nowowiejski Hospital in Warsaw. The HC group was recruited through advertisements on social media and matched according to education years. CSO+: M=43.8 (8.46), CSO-: M=36.56 (8.8), HC: M=32.24 (7.85)	An affective Go/NoGo task in a block design appropriate for a functional magnetic resonance imaging (fMRI) experiment. This task comprised the following conditions: Negative Go, Negative NoGo and two corresponding conditions with neutral pictures (Neutral Go and Neutral NoGo). Each block started with a cue showing the block type (Go or NoGo) and comprised 14 trials. fMRI data analysis was to the dorsolateral prefrontal (DLPFC), orbitofrontal, and anterior cingulate cortices and the general linear approach in SPM12 software was used.	• The HC and CSO- groups, but not the CSO+ group, showed significantly slower reactions in negative blocks compared with neutral blocks. • Brain analysis revealed increased activation in the right dorsolateral prefrontal cortex during emotional interference contrast (Negative > Neutral) in the HC and CSO- groups; however, there was decreased activation in the CSO+ group. • In the CSO+ group, negative distractors did not increase cognitive control processes, which was observed in the CSO- and HC groups at the behavioral and neural levels.
13	Weidacker et al. (2022) (39)	Germany	Focused on interference inhibition and examined event-related functional magnetic resonance imaging (fMRI) data of three groups of men performing a color-word Stroop task: (1) pedophiles with a history of CSO, (2) pedophiles without a history of CSO and (3) non-pedophilic, non-offending healthy controls.	11 pedophiles with a history of CSO (P+CSO, n = 11), 8 pedophiles without a history of CSO (P–CSO, n = 8) and 10 non-pedophilic, non-offending healthy controls (HC, n = 10). Pedophilic participants were recruited via explicit online forums, the research groups’ website, via the Prevention Project Dunkelfeld and from correctional services in North Rhine-Westphalia, Germany (NeMUP). Non-pedophilic controls were recruited from the community via flyers and advertisements in public institutions. P+CSO: M=43.55 (11.58), P-CSO: M=33.25 (10.79), HC: M=37.70 (13.12)	Event-related functional magnetic resonance imaging (fMRI) performing a color-word Stroop task, presenting words in either congruent or incongruent color against a black background.	• On the behavioral level, P+CSO revealed increased Stroop interference as compared to P–CSO and HC.

### Critical appraisal of evidence

In accordance with scoping review methodology, no studies were excluded based on quality appraisal outcomes. Appraisal was conducted for both case-control and cross-sectional studies (please see **Tables S2**, **S3** in **Supplementary material**).

### Results of individual sources of evidence

Biomarkers associated with pedophilia were categorized into four domains: (1) genetic/epigenetic and neuroendocrinal (n=5), (2) physiological, including electroencephalogram (EEG), penile plethysmography (PPG), and eye-tracking (n=10), (3) cognitive/behavioral (n=13), and (4) neuroimaging/neurofunctional (n=22), including fMRI and PET scan. Of the total 39 studies reviewed, 13 studies utilized fMRI as part of their methodology. Among these, 11 studies reported statistically significant findings. A comprehensive summary of the identified biomarkers is presented in **Tables 1**–**4**.

#### Genetic, epigenetic and neuroendocrinal biomarkers

Three studies investigated genetic and/or epigenetic biomarkers in pedophilia (40–42). Preliminary findings suggested involvement of epigenetic serotonergic system dysregulation, specifically indicating downregulation with alterations in 5HT3A (increased methylation) and SLC6A4 (reduced methylation) in subjects with a pedophilic sexual preference. Studies on the dopaminergic system reported no significant associations (40, 41).

Genetic and neuroendocrine studies suggest a potential link between epigenetic alterations in the serotonergic and testosterone systems, with lower testosterone levels and signs of prenatal androgen exposure observed in pedophilic individuals.

Neuroendocrine markers were assessed in three studies (19, 20, 42), revealing lower mean testosterone levels in pedophilic individuals compared to controls and indications of elevated prenatal androgen exposure in child sexual offenders compared to non-offending pedophiles and controls. One study identified methylation of the androgen receptor gene, in conjunction with reduced peripheral testosterone levels (42).

#### Physiological biomarkers (EEG, PPG, eye tracking)

Ten studies assessed physiological biomarkers, including brain responses (EEG, PPG) and eye tracking (19, 20, 43–50), reporting various physical and neurophysiological anomalies. Among the physiological biomarkers explored in the literature, minor physical anomalies (MPAs) have been identified as potential neurodevelopmental markers of pedophilic interest. In a study by Dyshniku et al. (43), MPAs were assessed using the Waldrop Physical Anomaly Scale, which quantifies 18 specific anomalies, including fine electric hair, hair whorls, abnormal head circumference, epicanthus, malformed ears, furrowed or irregular tongue, high/steepled palate, curved fifth finger, single palmar crease, and various toe anomalies.

Findings indicated increased minor physical abnormalities, elevated craniofacial-to-peripheral anomalies ratio (43), shorter leg length (45), higher rates of non-right or ambiguous handedness (44), as well as attentional control and greater sexual arousal ratings and erectile responses to undressed versus clothed child (visual sexual stimuli), suggesting dysfunction in prefrontal cortex and error processing areas (19, 20).

Regarding eye-movement variables, findings included shorter entry time to child stimuli and longer relative fixation time on adult stimuli (46), reduced attentional control (47, 48), and impaired executive functioning on interference tasks (47).

#### Cognitive/behavioral biomarkers

Cognitive impairments, especially in memory and executive functions, are commonly observed in pedophilia, with specific differences found between child sexual offenders and non-offenders, as well as convicted versus non-convicted offenders.

Thirteen studies explored cognitive and behavioral biomarkers (16, 22, 23, 25, 26, 38, 39, 51–56). Most found significant impairments in IQ, memory (immediate and delayed), visuospatial/constructional abilities, processing speed (slower visual performance, slower reaction time, less accurate visual target discrimination, visual-motor integration, processing speed), and set-shifting and executive function (response inhibition, attention and impulse control) (16, 23, 38, 39, 51, 52, 54, 56). One study, however, reported better performance on verbal memory and visual discrimination in participants with pedophilia (54).

Moreover, four studies showed that pedophilic child sexual offenders compared to pedophilic non-child sexual offenders, had lower IQ, poorer inhibition ability, more working memory errors, greater risk-taking behavior and higher interference susceptibility (25, 26, 53, 55). One study reported differences between convicted and non-convicted child sexual offenders in sexual age orientation, offense patterns (number of delicts) and victim profiles (number of male victims, age of female victims) (22).

#### Neuroimaging/neurofunctional biomarkers

Neuroimaging studies highlighted significant structural and functional brain differences in pedophilia, with evidence of altered connectivity, volume reductions, and abnormal brain activation patterns, particularly in areas related to emotional regulation, cognitive control, and sexual behavior. Specifically, twenty-two studies applied neuroimaging techniques (16–29, 32–39). Structural findings included reduced intracranial volume, cortical thickness and volume of white matter, smaller hippocampi and nuclei accumbens and abnormalities in cortical surface area (16, 26). Diffusion tensor imaging (DTI) studies found elevated fractional anisotropy (17), increased amygdala (prefrontal cortex connectivity) supporting models of disinhibited aberrant sexual impulses and impaired higher-order cognitive control (33), while one study reported lower fractional anisotropy, particularly in the corpus callosum (26).

The fMRI results showed altered activation in response to visual child stimuli in regions, such as the right inferior temporal cortex, which are associated with sexual arousal (erection) (19, 20, 29). Other studies reported atypical activations in networks related to moral judgment, such as the left temporo-parietal-junction, left posterior insular cortex, posterior cingulate gyrus and precuneus (27), while others in networks related to motivating behaviors, such as nurturing (left anterior insular cortex, supplementary motor cortex, and dorsolateral prefrontal areas) (32). Dysregulation functional connectivity with the default mode network, including deactivation of some regions, and regional differences (increases and decreases) in the frontoparietal network were also reported (17, 18, 23). Additionally, GABA reductions in the dorsal anterior cingulate cortex, a region linked to executive control process, were associated with low self-control and ADHD-like symptoms (34). However, not all studies found significant differences between pedophilic and teleiophilic individuals (21, 22, 25, 28).

Furthermore, comparative imaging studies of offending (P+CSO) versus non-offending (P-CSO) pedophiles revealed reduced resting-state functional connectivity in both the default mode and limbic network in P+CSO, especially in the left amygdala and orbitofrontal and anterior prefrontal regions (24). Additional findings in P+CSO group included smaller hypothalamus per side (37) and reduced activity in the a) medial parietal cortex, including the left caudal posterior cingulate and left superior frontal cortex (25), b) right dorsolateral prefrontal cortex (indicating impaired emotional regulation) (38), and c) left precuneus. Conversely, P−CSO individuals demonstrated increased activation and enhanced connectivity between regions implicated in cognitive empathy (left anterior cingulate cortex) (36). Additionally, P+CSO group showed a significantly reduced relative gray matter volume in the right temporal pole (35), and higher interference susceptibility, as well as hyperactivation in the left superior parietal cortex and precentral gyrus/supramarginal gyrus, therefore indicating potential difficulties to reallocate attention away from dominant tendencies (39).

### Synthesis of results

Across domains, the reviewed literature indicated that multiple biomarkers may contribute to the identification and understanding of pedophilia. Genetic and epigenetic studies suggest involvement of neuroendocrine markers, i.e., serotonergic and testosterone systems. Physiological markers, including EEG, PPG, and eye-tracking, showed deviations in physical development, attentional control, and sexual arousal responses. Cognitive findings point to impairments in memory and executive function, with distinctions observed between offending and non-offending groups. Neuroimaging studies consistently highlighted structural and functional brain abnormalities in regions associated with emotional regulation, cognitive control, and sexual behavior. A detailed summary of study findings is provided in **Tables 1**–**4**.

## Discussion

To our knowledge this is the first comprehensive review to synthesize existing data on biomarkers associated with pedophilia. By mapping findings across genetic, physiological, cognitive, and neuroimaging domains, the present review presented a structured overview of the current scientific landscape and identified important gaps that hinder the development of reliable diagnostic or risk-related biomarkers in the field. Overall, the present findings highlighted both the promise and complexity of identifying neurobiological correlates in pedophilia, while revealing the need for coordinated, ethically sound, and methodologically rigorous further research.

Among the four biomarker categories reviewed herein, neuroimaging and neurofunctional studies dominated the research field. These consistently reported alterations in brain structure and function, including reduced intracranial volume, cortical surface area abnormalities, and white matter volume differences. Functional neuroimaging findings reported divergent brain activation patterns to visual sexual stimuli, altered processing in moral reasoning and nurturing-related brain regions, and disrupted connectivity within the default mode network and frontoparietal networks. Notably, differences in brain structure and function (functional connectivity, brain activation, and gray matter volume) observed between individuals with pedophilic disorder who committed offenses and those who did not, suggesting potential neural correlates of behavioral control or risk factors for offending.

Cognitive and behavioral studies revealed a profile marked by deficits in IQ, memory, attention, and executive functioning. Lower IQ (working memory), greater susceptibility to interference and risk-taking behavior, and poorer inhibition performance were more pronounced among individuals with pedophilic disorders who committed child sexual offences, compared to those who did not. These impairments may point to broader neuropsychological vulnerabilities related to impulse regulation and decision-making. However, isolated findings of intact or even enhanced verbal memory suggest heterogeneity within this population, emphasizing the need for nuanced subgroup analyses.

Physiological biomarkers identified in EEG, plethysmography and eye-tracking studies showed promising data in identifying attentional and arousal patterns specific to pedophilic interests. Reported differences included increased physical abnormalities, atypical handedness, and shorter leg length. These may reflect underlying neurodevelopmental anomalies. In addition, eye-tracking data indicate shorter gaze latency toward child stimuli and reduced attentional control, while EEG findings point to diminished early frontal responses and altered error processing—both suggestive of atypical neural reactivity during sexual or executive tasks.

Genetic, epigenetic, and neuroendocrine biomarkers remained understudied, though early evidence suggested potential epigenetic alterations in the serotonergic and testosterone systems, while findings related to the dopaminergic system remained inconclusive. Specifically, findings included reduced peripheral testosterone levels, signs of elevated prenatal androgen exposure, and methylation changes in genes related to serotonin transport. While preliminary, these results suggest that hormonal and epigenetic factors may contribute to the biological underpinnings of pedophilia.

Despite these advances, no single biomarker demonstrated sufficient sensitivity or specificity to support clinical diagnostic use. Instead, findings across domains may be best understood as potential contributors to risk stratification, pathophysiological insight, or future treatment personalization. Overall, pedophilia is a complex and heterogeneous condition, varying widely in age of attraction, degree of behavioral control, comorbidity profiles, and offending history. These individual differences seem to contribute to inconsistent biomarker findings and complicate efforts to generalize across study populations.

### Limitations

This review is subjected to several methodological constraints. While the decade-based publication restriction was applied to capture up-to-date biomarker research, it may have excluded foundational or earlier relevant studies. Moreover, excluding non-English literature may have limit the scope and introduce a potential language bias. Additionally, reliance on title and abstract screening during the selection of sources of evidence may have led to the inadvertent exclusion of relevant studies where biomarker relevance was only evident in full-text content.

The reviewed studies, also, present numerous limitations. Firstly, the study samples consisted exclusively of males, which limits the generalizability of the findings to women. Additionally, while some studies included relevant confounders such as sex, age, IQ/education, psychiatric morbidity, left-handedness, psychotropic medication, drug/alcohol use, and sexual orientation, not all studies controlled for these variables comprehensively. This lack of control for confounders can introduce bias and affect the validity of the results. Moreover, the included studies varied widely in methodology and reporting standards. This heterogeneity posed challenges for consistent data extraction and synthesis. Specifically, there was significant heterogeneity in the diagnosis of pedophilia and inconsistencies regarding the operational definitions of pedophilia across studies (e.g., DSM-5 criteria vs. ICD-11 or offense-based classification), which can lead to variations in the findings.

Differences were also noted in relation to neuroimaging protocols, and varied tasks used in cognitive or physiological assessments. The heterogeneity in study samples, including variations in the severity and characteristics of pedophilic behavior, further complicates the interpretation of results. Many studies also had small sample sizes, which reduces the statistical power and the ability to detect significant differences or associations. Furthermore, specific biomarkers were not consistently investigated across studies, and some biomarkers were not examined at all, leading to gaps in the understanding of the neurobiological underpinnings of pedophilia.

### Future directions

These limitations highlight the need for more rigorous and standardized research methodologies in future studies to enhance the robustness and applicability of the results. By addressing these limitations, future research can enhance the robustness, validity, and applicability of findings, ultimately contributing to a better understanding of pedophilia and possibly informing effective interventions. Collaborative efforts across disciplines are essential, with adequate sample size in well characterized populations has the potential to advance biomarker research and improve diagnostic accuracy, treatment outcomes, and risk assessment for individuals with pedophilic disorders. Crucially, longitudinal designs and replication studies are needed to test the reliability and prognostic value of proposed biomarkers over time. Ultimately, for any biomarker to be used clinically, it must demonstrate a statistically significant relationship to a defined clinical endpoint, a causal or mechanistic link, and adequate sensitivity and specificity, validated through large-scale, preregistered trials.

The use of integrative methods using multimodal approaches that combine cognitive, neuroimaging, physiological, and genetic/epigenetic measures to provide a holistic understanding of pedophilia would be valuable. Specifically, future research should aim for larger, well-characterized samples, preferably through multicenter collaboration. Standardized diagnostic criteria and consistent measurement protocols would improve replicability. Applying the Research Domain Criteria (RDoC) approach may yield novel insights and facilitate biomarker development, also allowing researchers to study pedophilic traits dimensionally across multiple neurobiological and behavioral domains, rather than solely within categorical diagnoses. Although none of the current biomarkers are ready for diagnostic implementation, the integration of multiple biomarker modalities—such as combining neuroimaging, cognitive, physiological, and genetic/epigenetic data—may enhance the predictive accuracy of risk assessment tools and contribute to a more precise understanding of the disorder. Advanced analytical techniques, including machine learning and multivariate pattern analysis, could help identify meaningful subtypes and predict clinically relevant outcomes. Importantly, any translational effort must be grounded in strong ethical principles. The use of biomarkers to identify pedophilic interests raises complex questions regarding consent, privacy, potential stigma, and the risk of misuse. As such, biomarker research must prioritize not only scientific validity but also legal, clinical, and human rights safeguards.

## Conclusion

This review offers the first comprehensive synthesis of biomarkers in pedophilia, identifying several promising biological systems potentially implicated in the pathophysiology of the disorder. While significant strides have been made, especially in neuroimaging, major methodological, ethical, and interpretive challenges remain. A multimodal, interdisciplinary, and ethically grounded approach will be critical to advancing this complex and sensitive field.

## Data Availability

The original contributions presented in the study are included in the article/**Supplementary Material**. Further inquiries can be directed to the corresponding author.
