# Supplementary material for: Mapping current research on biomarkers associated with the diagnosis of pedophilia: a scoping review
Source: Front Psychiatry. 2025 Oct 16;16:1627198. doi: 10.3389/fpsyt.2025.1627198 (PMC12574379; doi:10.3389/fpsyt.2025.1627198)
Supplement: Supplementary file 1 [file Supplementaryfile1.docx]

**Supplementary material**

Table S1: Preferred Reporting Items for Systematic reviews and Meta-Analyses extension for Scoping Reviews (PRISMA-ScR) Checklist

| **SECTION** | **ITEM** | **PRISMA-ScR CHECKLIST ITEM** | **REPORTED IN SECTION** |
| --- | --- | --- | --- |
| **TITLE** | | | |
| Title | 1 | Identify the report as a scoping review. | Title page |
| **ABSTRACT** | | | |
| Structured summary | 2 | Provide a structured summary that includes (as applicable) background, objectives, eligibility criteria, sources of evidence, charting methods, results, and conclusions that relate to the review questions and objectives. | Abstract |
| **INTRODUCTION** | | | |
| Rationale | 3 | Describe the rationale for the review in the context of what is already known. Explain why the review questions/objectives lend themselves to a scoping review approach. | Introduction /  Research questions |
| Objectives | 4 | Provide an explicit statement of the questions and objectives being addressed with reference to their key elements (e.g., population or participants, concepts, and context) or other relevant key elements used to conceptualize the review questions and/or objectives. |  |
| **METHODS** | | | |
| Protocol and registration | 5 | Indicate whether a review protocol exists; state if and where it can be accessed (e.g., a Web address); and if available, provide registration information, including the registration number. | Protocol and registration |
| Eligibility criteria | 6 | Specify characteristics of the sources of evidence used as eligibility criteria (e.g., years considered, language, and publication status), and provide a rationale. | Eligibility criteria |
| Information sources* | 7 | Describe all information sources in the search (e.g., databases with dates of coverage and contact with authors to identify additional sources), as well as the date the most recent search was executed. | Information sources |
| Search | 8 | Present the full electronic search strategy for at least 1 database, including any limits used, such that it could be repeated. | Search strategy |
| Selection of sources of evidence† | 9 | State the process for selecting sources of evidence (i.e., screening and eligibility) included in the scoping review. | Selection of sources of evidence |
| Data charting process‡ | 10 | Describe the methods of charting data from the included sources of evidence (e.g., calibrated forms or forms that have been tested by the team before their use, and whether data charting was done independently or in duplicate) and any processes for obtaining and confirming data from investigators. | Data charting process |
| Data items | 11 | List and define all variables for which data were sought and any assumptions and simplifications made. | Eligibility criteria |
| Critical appraisal of individual sources of evidence§ | 12 | If done, provide a rationale for conducting a critical appraisal of included sources of evidence; describe the methods used and how this information was used in any data synthesis (if appropriate). | Critical appraisal of individual sources of evidence |
| Synthesis of results | 13 | Describe the methods of handling and summarizing the data that were charted. | Synthesis of results |
| **RESULTS** | | | |
| Selection of sources of evidence | 14 | Give numbers of sources of evidence screened, assessed for eligibility, and included in the review, with reasons for exclusions at each stage, ideally using a flow diagram. | Selection of sources of evidence/Flowchart |
| Characteristics of sources of evidence | 15 | For each source of evidence, present characteristics for which data were charted and provide the citations. | Characteristics of sources of evidence |
| Critical appraisal within sources of evidence | 16 | If done, present data on critical appraisal of included sources of evidence (see item 12). | Critical appraisal of evidence/Table S2/Table S3 |
| Results of individual sources of evidence | 17 | For each included source of evidence, present the relevant data that were charted that relate to the review questions and objectives. | Results of individual sources of evidence/  Genetic, epigenetic and neuroendocrinal biomarkers/  Physiological biomarkers (EEG, PPG, eye tracking)/  Cognitive/behavioral biomarkers/  Neuroimaging/neurofunctional biomarkers |
| Synthesis of results | 18 | Summarize and/or present the charting results as they relate to the review questions and objectives. | Synthesis of results |
| **DISCUSSION** | | | |
| Summary of evidence | 19 | Summarize the main results (including an overview of concepts, themes, and types of evidence available), link to the review questions and objectives, and consider the relevance to key groups. | Discussion |
| Limitations | 20 | Discuss the limitations of the scoping review process. | Limitations |
| Conclusions | 21 | Provide a general interpretation of the results with respect to the review questions and objectives, as well as potential implications and/or next steps. | Conclusions |
| **FUNDING** | | | |
| Funding | 22 | Describe sources of funding for the included sources of evidence, as well as sources of funding for the scoping review. Describe the role of the funders of the scoping review. | Funding |

JBI = Joanna Briggs Institute; PRISMA-ScR = Preferred Reporting Items for Systematic reviews and Meta-Analyses extension for Scoping Reviews.

* Where *sources of evidence* (see second footnote) are compiled from, such as bibliographic databases, social media platforms, and Web sites.

† A more inclusive/heterogeneous term used to account for the different types of evidence or data sources (e.g., quantitative and/or qualitative research, expert opinion, and policy documents) that may be eligible in a scoping review as opposed to only studies. This is not to be confused with *information sources* (see first footnote).

‡ The frameworks by Arksey and O’Malley (6) and Levac and colleagues (7) and the JBI guidance (4, 5) refer to the process of data extraction in a scoping review as data charting*.*

§ The process of systematically examining research evidence to assess its validity, results, and relevance before using it to inform a decision. This term is used for items 12 and 19 instead of "risk of bias" (which is more applicable to systematic reviews of interventions) to include and acknowledge the various sources of evidence that may be used in a scoping review (e.g., quantitative and/or qualitative research, expert opinion, and policy document).

*From:* Tricco AC, Lillie E, Zarin W, O'Brien KK, Colquhoun H, Levac D, et al. PRISMA Extension for Scoping Reviews (PRISMAScR): Checklist and Explanation. Ann Intern Med. 2018; 169:467–473. [doi: 10.7326/M18-0850](http://annals.org/aim/fullarticle/2700389/prisma-extension-scoping-reviews-prisma-scr-checklist-explanation).

Table S2: JBI Critical Appraisal Checklist for Case Control Studies

| No | **Case Control Studies/JBI tool** | **C1** | **C2** | **C3** | **C4** | **C5** | **C6** | **C7** | **C8** | **C9** | **C10** |
| --- | --- | --- | --- | --- | --- | --- | --- | --- | --- | --- | --- |
| 1 | Abé et al., 2021 | Yes | Yes | Yes | Yes | Yes | Yes | Yes | Yes | N/A | Yes |
| 2 | Cantor et al., 2015 | Yes | Yes | Yes | Yes | Yes | Yes | Yes | Yes | N/A | Yes |
| 3 | Cantor et al., 2016 | Yes | Yes | Yes | Yes | Yes | Yes | Yes | Yes | N/A | Yes |
| 4 | Cazala et al., 2019 | Yes | Yes | Yes | Yes | Yes | Yes | Yes | Yes | N/A | Yes |
| 5 | Fazio et al., 2017 | Yes | Yes | Yes | Yes | Yes | No | No | Yes | N/A | Yes |
| 6 | Franke et al., 2019 | No | No | No | No | No | No | No | Yes | N/A | No |
| 7 | Gerwinn et al., 2015 | Yes | Yes | Unclear | Yes | No | Unclear | Unclear | Yes | N/A | Yes |
| 8 | Habermeyer et al., 2013 | Yes | Yes | Yes | Yes | Unclear | Yes | Unclear | Yes | N/A | Yes |
| 9 | Jahn et al., 2022 | Unclear | Unclear | Yes | Yes | Yes | Yes | Yes | Yes | N/A | Yes |
| 10 | Jakubczyk et al., 2017 | Unclear | Unclear | Unclear | Yes | Unclear | Unclear | Unclear | Yes | N/A | Unclear |
| 11 | Kärgel et al., 2015 | Yes | Yes | Yes | Yes | Unclear | Yes | Yes | Yes | N/A | Yes |
| 12 | Kruger et al., 2019 | Yes | Yes | Yes | Yes | Yes | Yes | Yes | Yes | N/A | Yes |
| 13 | Kärgel et al., 2017 | Yes | Yes | Unclear | Yes | Unclear | Yes | Yes | Yes | N/A | Yes |
| 14 | Picard et al., 2023 | Yes | Yes | Yes | Yes | Yes | No | No | Yes | N/A | Yes |
| 15 | Weidacker et al., 2022 | Yes | Yes | Yes | Yes | Yes | Yes | Yes | Yes | N/A | Yes |
| 16 | Schuler et al., 2022 | Yes | Yes | Yes | Yes | Yes | Yes | Yes | Yes | N/A | Yes |
| 17 | Szczypinski et al., 2022 | Unclear | Unclear | Yes | Yes | Yes | Yes | Yes | Yes | N/A | Yes |
| 18 | Rosburg et al., 2021 | Yes | Yes | No | Yes | Yes | Yes | Yes | Yes | N/A | Yes |
| 19 | Ristow et al., 2018 | Yes | Yes | Yes | Yes | Yes | Yes | Yes | Yes | N/A | Yes |
| 20 | Lett et al., 2018 | No | No | Yes | Yes | Yes | Yes | Yes | Yes | N/A | Yes |
| 21 | Massau et al., 2017 | Yes | Yes | Yes | No | Yes | Yes | Yes | No | N/A | Yes |
| 22 | Massau et al., 2017 | Yes | Yes | Yes | Yes | Yes | Yes | Yes | Yes | N/A | Yes |
| 23 | Ponseti et al., 2014 | Unclear | Unclear | Unclear | Unclear | Unclear | No | No | Unclear | N/A | Unclear |
| 24 | Storch et al., 2023 | No | No | Yes | Yes | Yes | Yes | Yes | Yes | N/A | Yes |
| 25 | Ponseti et al., 2016 | Unclear | Yes | Unclear | Yes | Yes | Yes | Yes | Unclear | N/A | Unclear |
| 26 | Suchy et al., 2014 | Yes | Yes | Yes | Yes | Yes | Yes | Yes | Yes | N/A | Yes |
| 27 | Schiffer et al., 2017 | Yes | Yes | Yes | Yes | Yes | Yes | Yes | Yes | N/A | Yes |
| 28 | Rosburg et al., 2018 | Yes | No | No | Yes | Yes | Yes | Yes | Yes | N/A | Yes |
| 29 | Azizian et al., 2016 | Yes | Yes | Yes | Yes | Yes | No | Unclear | Yes | N/A | Yes |
| 30 | Fonteille et al., 2019 | Yes | Yes | Yes | Yes | Yes | Yes | Yes | Yes | N/A | Yes |
| 31 | Knott et al., 2016 | Yes | Yes | Yes | Yes | Yes | Yes | Yes | Yes | N/A | Yes |
| 32 | Fromberger et al., 2013 | Yes | Yes | Yes | Yes | Yes | Yes | Yes | Yes | N/A | Yes |
| 33 | Jordan et al., 2016 | No | Yes | Yes | Yes | Yes | Yes | Yes | Yes | N/A | Yes |
| 34 | Jordan et al., 2018 | No | No | Yes | Yes | Yes | Yes | Yes | Yes | N/A | Yes |
| 35 | Gibbels et al., 2019 | Unclear | Yes | Yes | Yes | Yes | No | Yes | Yes | N/A | Yes |
| 36 | Ponseti et al., 2018 | Yes | Yes | Yes | Yes | Yes | Yes | Yes | Yes | Yes | Yes |
| 37 | Popovic et al., 2023 | Yes | Yes | Yes | Yes | Yes | Yes | Yes | Yes | N/A | Yes |

C1: Were the groups comparable other than the presence of disease in cases or the absence of disease in controls?

C2: Were cases and controls matched appropriately?

C3: Were the same criteria used for identification of cases and controls?

C4: Was exposure measured in a standard, valid and reliable way?

C5: Was exposure measured in the same way for cases and controls?

C6: Were confounding factors identified?

C7: Were strategies to deal with confounding factors stated?

C8: Were outcomes assessed in a standard, valid and reliable way for cases and controls?

C9: Was the exposure period of interest long enough to be meaningful?

C10: Was appropriate statistical analysis used?

Table S3: JBI Critical Appraisal Checklist for analytical Cross-Sectional Studies

| No | **Cross-sectional studies/JBI tool** | **C1** | **C2** | **C3** | **C4** | **C5** | **C6** | **C7** | **C8** |
| --- | --- | --- | --- | --- | --- | --- | --- | --- | --- |
| 1 | Dyshniku et al., 2015 | Yes | No | Yes | Yes | No | No | Yes | N/A |
| 2 | Fazio et al., 2014 | No | Unclear | Unclear | No | No | No | Unclear | Yes |

C1: Were the criteria for inclusion in the sample clearly defined?

C2: Were the study subjects and the setting described in detail?

C3: Was the exposure measured in a valid and reliable way?

C4: Were objective, standard criteria used for measurement of the condition?

C5: Were confounding factors identified?

C6: Were strategies to deal with confounding factors stated?

C7: Were the outcomes measured in a valid and reliable way?

C8: Was appropriate statistical analysis used?
